# Adolescent girls trapped in early marriage social norm in rural Ethiopia: A vignette-based qualitative exploration

**DOI:** 10.1371/journal.pone.0263987

**Published:** 2022-02-17

**Authors:** Dagmawit Tewahido, Alemayehu Worku, Amare W. Tadesse, Hanna Gulema, Yemane Berhane

**Affiliations:** 1 Nutrition and Behavioral Sciences Department, Addis Continental Institute of Public Health, Addis Ababa, Ethopia; 2 Epidemiology and Biostatistics Department, Addis Continental Institute of Public Health, Addis Ababa, Ethopia; 3 Infectious Disease Epidemiology, London School of Hygiene and Tropical Medicine, London, England; 4 Global Health and Health Policy Department, Addis Continental Institute of Public Health, Addis Ababa, Ethopia; Kyushu University, JAPAN

## Abstract

**Background:**

Early marriage is not uncommon in Ethiopia, particularly for adolescent girls in rural settings. Social norms are among the factors believed to perpetuate early marriage practices. This qualitative study explores social norms surrounding adolescent girls’ marriage practices in West Hararghe, Ethiopia.

**Methods:**

This study used the qualitative inquiry method to explore social norms in rural Ethiopia. Focus group discussions were conducted with purposively sampled married and unmarried adolescent girls, adolescent boys, and parents. A total of 158 individuals participated in the study, comprising 95 adolescents and 63 parents. Data were collected using locally developed vignettes. A thematic framework analysis approach using the Social Norms Analysis Plot (SNAP) was employed to diagnose and understand social norms.

**Results:**

Adolescent girls’ marriage was found to be mainly influenced by their peers who conform to prevailing social norms. Marrying one’s first suitor was considered an opportunity not to be missed and a symbol of good luck. Relatives, neighbors, and marriage brokers facilitate adolescent girls’ marriage in accordance with the local social norms. Girls usually accept the first marriage proposal regardless of their age, and they are highly expected to do so by their peers, parents, and influential others. Exceptions from the early marriage social norm include adolescent girls determined to continue their education and those having supportive teachers.

**Conclusions:**

In this study context, social norms strongly encourage early marriage and are mainly perpetuated by peers of adolescent girls and influential adults. A strong determination to continue education on the part of girls, strong school performance, and supportive schoolteachers are important conditions for circumventing social norms on early marriage. As social norms evolve slowly, we recommend periodical assessment in order to develop locally appropriate interventions against early marriage.

## Introduction

Globally, one out of nine girls is married before 15 years of age [1]. Ethiopia is among the top five countries where early marriage is widely practiced [2]. Girls who marry at a young age face negative consequences in their life course due to sexual reproductive health issues, failure to achieve better education, economic disadvantages, and compromised agency [3]. Ensuring girls grow up protected from early marriage and other exploitation is considered key to societal development beyond just upholding the basic rights of the girls [4, 5].

Although many factors influence early marriage in low-income settings, social norms enforce behaviour/practice more than any other social construct [6, 7]. Social norms, according to Cristina Bicchieri, are described as unwritten rules of behaviour that individuals choose to conform to on the condition that they believe that most people in their reference group also conform to them (empirical expectation) and that most people in their reference group expect they ought to conform (normative expectation), so their preferences are largely dependent on their perceptions of others’ deeds and expectations, which is referred as conditional preferences [8]. Reference groups are individuals/groups that the index person/group (the person/group who is studied regarding the specific social norm) has high regard for and whose thoughts and judgments matter most [9]. Reference groups to a particular index person and to a particular behaviour can be different in different contexts.

Unfavorable social norms enhance girls’ vulnerability to early marriage [10]. Unfavorable social norms also tend to suppress young girls’ aspirations and opportunities to be independent people with greater autonomy; as a result, young girls may become wives, mothers, and caretakers of their newly established families before they physically and mentally mature into full adults [11, 12].

Traditional marriage practices in which girls get married at a very young age remain prevalent in Ethiopia, particularly in rural settings [11], despite the legal restrictions on marriage below the age of 18 years [13]. According to Article 7.1 of Ethiopian family law, neither a man nor a woman who has not attained the full age of 18 years shall undertake marriage [14]. However, implementation of the law has not been fully enforced in rural areas due to prevailing social norms. Yet, there is a paucity of information on social norms surrounding early marriage in Ethiopia, especially considering the wide range of contextual variations within the same country [5]. Understanding prevailing social norms that influence early marriage decisions is key to designing and implementing interventions that promote the health and well-being of young adolescent girls.

The pressure to be married at a young age seriously affects the physical and mental health of girls, both immediately after marriage and in the future [15]. Nonetheless, social norms are sustained because many fall into the trap of living up to others’ expectations regardless of individual motives to conform to certain practices [16].

Thus, this study aims to explore the prevailing social norms around adolescent girls’ early marriage practices in rural West Hararghe districts in Ethiopia. A qualitative method was chosen for this study because of the sensitivity and deep-rooted nature of the studied phenomena: social norms around early marriage. In addition, highly contextual and behavioural characteristics are best captured by the use of qualitative approaches.

## Methods

### Study area

The study was conducted in four districts of the rural West Hararghe zone in Oromia Regional State, Ethiopia. The livelihood in the area is mainly dependent on subsistence agriculture. The area is known to be prone to cyclic drought and is chronically food insecure. The economy is largely subsistence, and households are often seriously affected by any negative climatic or human-made calamities. The majority of the residents are followers of the Islam religion, and the local language is *Afaan Oromo*. Primary schools are available in almost all residential neighborhoods; however, high schools may not always be available in close vicinity, and young adolescent girls need to travel to town or stay in town by themselves to attend high school. The political change that engulfed the whole country while we conducted the fieldwork of this study has negatively affected the quality and accessibility of social services, including the education and health sectors. It was not uncommon for schools to close time and again during the study period due to the widespread popular political movements.

This study was part of a large project that aimed to improve adolescent girls’ reproductive health and nutrition through structural solutions. The interventions were implemented by another independent non-governmental organization. A mix of qualitative and quantitative studies was conducted by an independent research team alongside the interventions to inform the implementation of interventions and decision-making throughout the project life.

### Study design

This paper utilized the qualitative data generated by the parent project. The analysis was planned from the inception of the project.

### Study participants

The study participants for the focus group discussions (FGDs) were organized into two main groups; one group served as reference for unmarried and the other for married adolescent girls. Group 1 comprised adolescents aged 13–17 years, further grouped into three homogenous categories of married adolescent girls, unmarried adolescent girls, and unmarried adolescent boys; Group 2 comprised parents of adolescent girls, with separate groups for mothers/women and fathers/men. Participants of the FGDs were recruited for the study using a purposive sampling strategy. The selection/inclusion criteria include being a permanent resident in the study area, being well acquainted with the culture of the society, and willingness to participate in the study. Participants were identified with the help of community health workers in the community and teachers in schools. Then, additional participants were recruited by the adolescents. Individuals who were not willing were excluded from the selection. Theoretical saturation was declared when no new information was forthcoming to further enrich the analysis framework as described assessed by Graneheim and Lundman [17].

### Study tools

We conducted vignette-based FGDs to explore social norms around young adolescent girls’ marriage. The vignettes portrayed hypothetical scenarios to enable participants to envision a situation in their own context. Qualitative exploration of social norms using vignettes has proven to be extremely useful [18]. The FGDs typically start with participants listening to and discussing a vignette, then continuing the discussion on related issues using a pre-prepared discussion guide. This technique is believed to better elicit people’s actual behaviour and their perceptions when dealing with very sensitive issues such as early marriage that are culturally endorsed but legally prohibited [19].

### How the vignettes were developed

The vignettes for this study were developed in a series of processes. Initially, we conducted a mini formative study involving adolescents, parents, husbands, and in-laws to identify marriage social norms reference groups and related sanctions relevant to the local context. Then, the research team, in a collaborative process, drafted multiple vignettes employing different scenarios and validated them by involving local experts and people targeted for the research. The validation process helped us to refine the vignettes, especially in using locally appropriate language and improving the scenarios cast in the stories. Finally, we chose two distinct vignettes, one targeted at adolescents (unmarried girls, unmarried boys, and married girls) and the other targeted at parents of adolescent girls (mothers and fathers). The refined vignettes were used in the main study. The whole process and the prolonged time spent in the field helped the research team to better understand the context.

The vignettes included a short story followed by five questions which represent empirical expectations (what most people do in the scenario depicted in the story), normative expectations (what most other people expect the character to do in the depicted scenario), sanctions (consequences for defying expectations), sensitivity to sanctions (how seriously the sanctions would affect the character in the scenario), and conditions (exceptions from being sanctioned). The vignettes for each group are provided as a supplement file (Vignette based study tools in S1 Appendix).

### Data collection

The FGDs were conducted in a private space, mostly in a local health post compound. No one, including the staff of the health post, was allowed to access the discussion area. Participants were comfortably seated and were provided refreshments. Each FGD was conducted by two research assistants (RAs) who had master’s level educations and had five days of training specifically for this study. One of the RAs served as a moderator, and the second actively took notes. Most FGD sessions lasted between 95 and 120 minutes. The FGDs were conducted using the local language, *Afaan Oromo*; both research assistants were fluent in the language. At the end of each discussion, the research assistants went over the main points raised and confirmed with the discussants that their points had been captured accurately. Discussions were digitally recorded with the consent of participants. At the end of each data collection day, the research assistants and principal investigators had a debriefing session to reflect on the conducted discussions regarding the main issues raised as well as areas that brought challenges and insights. This launched the preliminary analysis process and continued until the data collection was completed.

### Data analysis

All digitally recorded FGDs were transcribed word-for-word in the *Afaan Oromo* language by the same research assistants who conducted the FGDs. Then, the materials were translated into English for analysis. A thematic codebook was developed based on the five main themes in the vignettes using the Social Norms Analysis Plot (SNAP) [20]. The initial coding was done by the first author and was reviewed and agreed upon by all authors. The constant comparison technique was used for exploring similarities and differences across respondent types [21]. We used the free OpenCode qualitative computer software [22] developed by Umea University in Sweden for managing, coding, and analyzing transcribed data. Then data analysis was mainly done based on the interpretative approach described by Rabiee [23]. The analysis framework involves social norm inquiries along five items: empirical expectations, normative expectations, sanctions, sensitivity to sanctions, and exceptions. These inquiries were prepared in a sequential flow manner, where the vignette-driven data were analysed to diagnose the conditional preferences along with sanctions and their strength and, finally, exceptions from sanctions. Illustrative quotes were integrated into the narratives to link the audience with the study participants’ own descriptions.

In this study, we followed several procedures to ensure trustworthiness. The prolonged stay in the field and the active engagement of stakeholders at the design stage were critical for developing locally appropriate vignettes. The involvement of adolescents and parents of adolescents helped to triangulate findings from different perspectives. The use of FGDs as a data collection method also helped to confirm consensus in a way that served as a member check. We did peer debriefing with local experts by presenting the study findings in a workshop. The research team members had the cultural competence and linguistic ability to interpret the findings. The field research activities and the process were documented in detail to allow thick description (transferability and dependability). The authors reflected on the findings independently and as a group on several occasions to ensure appropriate interpretation of the findings and to delineate their own presuppositions in the process.

### Ethical considerations

The research protocol was approved by the Institutional Review Board (IRB) of the Addis Continental Institute of Public Health (Ref No. ACIPH/IRB/005/2016). Informed verbal consent was obtained from all study participants after the study procedures and their rights were explained to them. For unmarried participants below the age of 15 years, assent and parental/guardian consent were obtained. Married adolescents were able to give consent according to the national ethical guideline, which was approved by IRB. All interviews took place in private spaces. Access to the original data was limited to only the study researchers. All individual identifiers were removed from all records to protect confidentiality.

## Results

A total of 20 FGDs were conducted for this study, and a total of 158 individuals participated: 32 unmarried adolescent girls, 32 unmarried adolescent boys, 30 married adolescent girls, 32 mothers of adolescent girls, and 32 fathers of adolescent girls. The adolescents’ ages ranged between 13 and 17, while mothers’ ages ranged from 30 to 50 years and fathers’ ages ranged from 32 to 70 years (Table 1).

**Table 1:** Focus group discussion participants distribution.

Target Group	No. of FGDs	No. of participants	Age of participants
**Unmarried adolescent girls recruited from schools**	4	32	13–16
**Unmarried adolescent boys recruited from schools**	4	32	14–17
**Married adolescent girls recruited from community**	4	30	14–17
**Mothers of adolescent girls recruited from community**	4	32	30–50
**Fathers of adolescent girls recruited from community**	4	32	32–70
**Total**	**20**	**158**	

The path to early marriage for young adolescent girls is commonly facilitated by local social norms that constitute expectations along with fear of and sensitivity to sanctions. The norms can shift in favour of delaying marriage with strong support for adolescent girls and parents.

**Fig 1:**
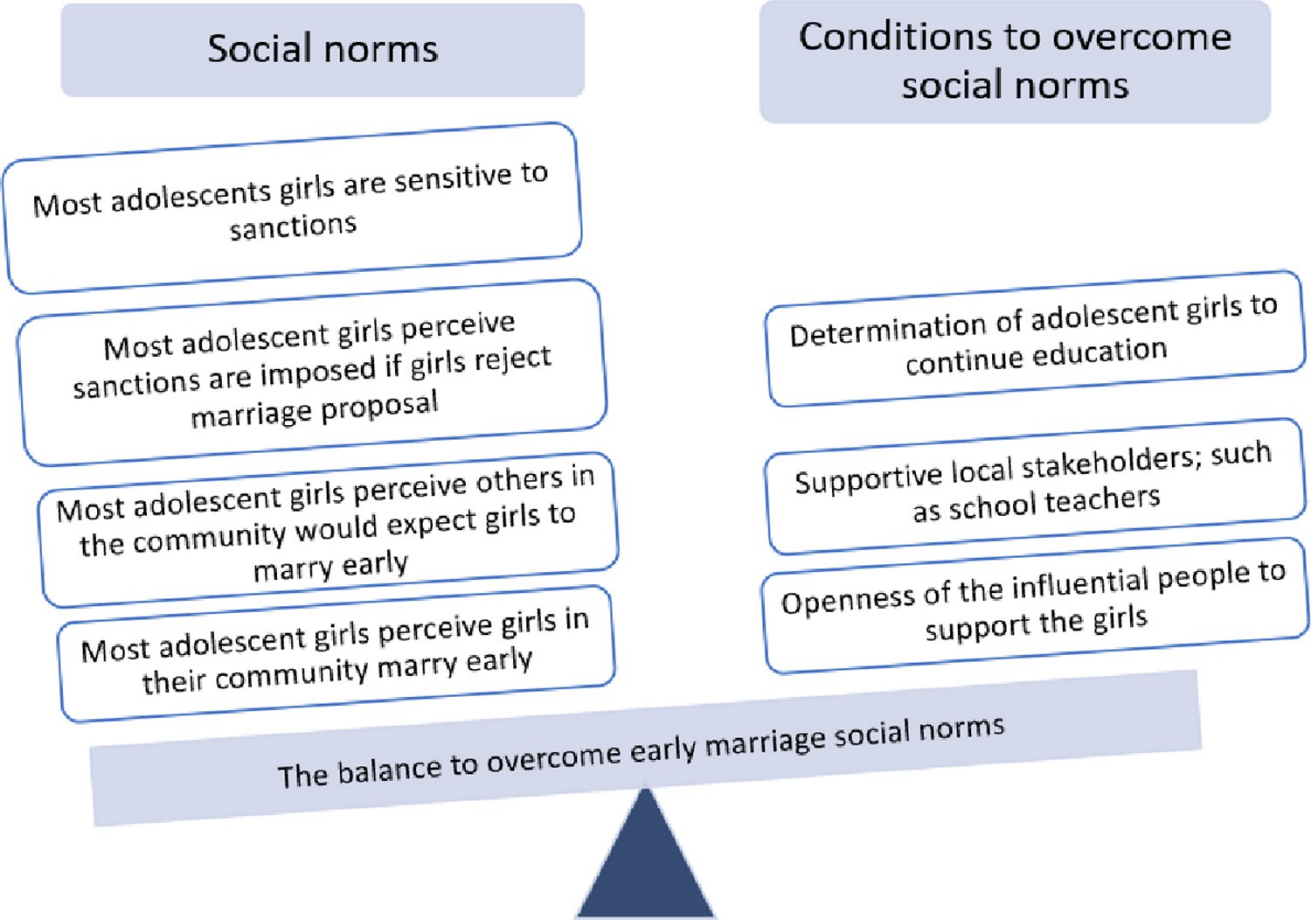
The balance to overcome social norms around early marriage in West Hararghe, Ethiopia.

Social norms can be subdued if conditions to overcome them outweigh their strength; in the study setting, the determination of the girl to continue her education and the willingness of influential people to consider exceptions was important in overcoming social norms. (Fig 1).

The study findings related to the five thematic areas identified based on the framework used for analysis are presented below. The first two constructs of the social norms analysis are presented first as collective social norms.

### Empirical and normative expectations (conditional preferences) around early marriage

Empirical and normative expectations were largely dependent on conditional preferences. That means people opt for these practices based on the conditions of others peoples’ common practices as well as reactions to/opinions about their actions.

#### Empirical expectations of early marriage

In the study area, marrying at a very young age was not uncommon for adolescent girls. Most adolescent girl and boy participants thought most girls would marry by the age of 15 years if a suitor presented himself. Girls were thought unlikely to refuse marriage when the chance was presented, even if that meant dropping out of school.

*“Most adolescent girls would accept marriage proposal*. *… Refusing is like wasting their fate*, *they may not get the chance again*. *In addition*, *they may feel refusing would offend their friends* (those who facilitated the proposal).*”–*Unmarried Adolescent Girl, District 1

*“I would decide to get married if proposed to*. *… Education is for making a living; so is marriage*. *I will drop* (out of school) *and marry*.*”* Unmarried Adolescent Girl, District 3

Most married girl participants said they would do what most other girls are doing in the community and that peers highly influence marriage decisions:

*“I got married when I was in fourth grade and while I was still in school because most of my friends were getting married*…. *I became alone*. *Then*, *I said to myself*, *why would I go to school alone*? *… That is how I got married*.*”–*Married Adolescent Girl, District 2

Boys also mentioned that most girls in their area are inclined to marry when they are around 15 years of age. Most mothers also felt that other mothers would be compelled to marry off their daughters at around that age. Although marriage was regarded mainly as the girl’s decision, in reality, mothers are thought to be highly expected to influence that decision. Similarly, the majority of the participants in the fathers’ FGDs also expressed their expectations that mothers would encourage their daughters to marry at around age 15 years. They said the chances of getting married for a girl decrease as her age increases, as ‘she gets old’.

*“Most girls in this area normally marry somewhere between the age of 14 and 18 years*. *If a girl could not find a husband in that age range*, *she will be called ‘Haftuu’* (meaning unlovable*)*.”–Father of Adolescent Girl, District 2

#### Normative expectations of early marriage

Most of the participants said that girls are generally expected to accept a marriage proposal even if it may entail dropping out of school.

*“*… *once the fortune of marrying comes in life*, *girls should not refuse but immediately go for marriage*.*”–*Unmarried Adolescent Girl, District 4

*“…a girl has to accept* (the marriage proposal) *and get married*, *unless she wants to be called ‘Haftuu’ and risk to be unmarriageable*.*”–*Adolescent Boy, District 1

Most of the mother participants felt people generally expect mothers to encourage, even pressure, their daughters to marry early. Girls’ refusal to marriage is not acceptable. A few mothers, however, said that mothers should not persuade their daughters to enter a marriage if the girl does not want to. Most father participants also said mothers would be inclined to marry off their daughters once they’d received a marriage proposal.

*“If a girl reaches 15 years it is the right time for her to marry*. *We* (other mothers) *would say to her* (mother of adolescent girl), *‘please marry off your daughter*…. *Marry her off*, *because like khat* (a local stimulant plant), *girls are best while still fresh*.*’”–*Mother of Adolescent Girl, District 2

### Sanctions and sensitivity to sanctions

Several negative consequences were mentioned that adolescent girls and/or their mothers might be subject to if they did not comply with early marriage social norms. Some of these sanctions could be quite strong; thus, the girls and mothers of adolescent girls were highly sensitive about them. The more detailed findings of the sanctions and sensitivity to sanctions are described below.

#### Sanctions for refusing early marriage proposal

The study participants mentioned different sanctions that may be imposed on adolescent girls and their mothers for rejecting a marriage proposal. Sanctions are commonly imposed by peers (both of the adolescent girl and the mother) and other people in their reference groups. Married and unmarried adolescent girls revealed that if an adolescent girl refused marriage, she would be lonely and discriminated against. Her friends would not want to be with her. They would consider her indolent, foolish, and useless. Adolescent boys revealed that, apart from being rejected by her friends, the girl who refuses to marry may be forced into marriage by an abduction set up by the suitor and his friends.

*“Her friends will tirelessly try to influence her to accept the marriage*, *and if she refuses*, *they may even facilitate to get her abducted*.*”–*Adolescent Boy, District 2

Marriage brokers would bad-mouth a mother who failed to persuade her daughter to marry. Mothers would also be disgraced and regarded as irresponsible by other parents for not persuading their daughters to accept the marriage proposal.

*“We* (peer mothers) *try to convince the mother* (of the proposed-to girl), *saying*, *‘if you refuse now*, *you may be humiliated if your daughter elopes with the boy*, *which will be a big shame to you and your family*.*’ This is the culture here*.*”–*Mother of Adolescent Girl, District 3

Marriage brokers play an important role in persuading mothers to think of the marriage proposal as a chance not to be missed. The brokers would make a marriage refusal sound like a grave mistake and a chance that may not come again. Mothers would also be threatened with disgrace unless they persuaded their daughters to accept the marriage proposal. Participants of the fathers’ FGDs also said if a girl refused to marry, the mother would be blamed for not being strong enough to persuade her daughter. Most fathers thought a girl is mature enough at age 15 years to get married, and a marriage proposal around that age should not be turned down.

*“…people would say*, *she has to go*, *she has to marry*, *it is time* (she is already 15 years old).*”–*Father of Adolescent Girl, District 1

#### Sensitivity to sanctions imposed for not accepting early marriage proposal

Most adolescent girls and parents expressed considerable sensitivity to sanctions that might be imposed for not abiding by the local marriage social norms. Particularly, the fear of persistent insults, isolation, or defamation was very strong.

*“In our community marriage is highly respected and her* (the proposed-to adolescent girl) *interest in education would not matter much*. *So even if she is not interested to get married*, *people and peers put pressure on her to get married while somebody shows interest*.*”–*Adolescent Boy, District 4

The study participants indicated even if some girls want to avoid the common practice of marriage, the severity and the fear of sanctions commonly supersede avoidance, and the girls commonly agree to the proposed marriage. The potential sanctions from their peers were the most feared. Mothers, similarly, give in to their daughter’s marriage proposal due to fear of sanctions.

### Exceptions/Conditions to minimize sanctions

Participants of the study indicated there might be some conditions/exceptions for adolescent girls to refuse marriage, including demonstrable success in their education. A girl who refuses marriage can still be accepted if she can convince her parents she is performing highly in school and she has supportive schoolteachers. However, according to most married adolescent girl and adolescent boy participants, such chances are slim as the community values marriage much more than education. If an adolescent girl resorts to a trusted teacher or a school administrator who is pro girls’ education, then she may be able to escape marriage as the teacher might threaten to report the parents to legal authorities (early marriage is illegal in Ethiopia).

*“In reality*, *it is very uncommon that the girl will say no to a marriage proposal … but if she is very strong in her education*, *she may inform a responsible teacher in her school*. *He then may try to convince her family and also stop her friend from putting pressure on her*.*”–*Married Adolescent Girl, District 1

*“If she* (adolescent girl) *wants her refusal to marriage to be accepted*, *she should firmly say she wants to continue her education*. *But she cannot refuse by saying she is not of age*.*”–*Mother of Adolescent Girl, District 4

## Discussion

The result shows early marriage is a conditional preference, conforming to societal expectations, and thus a persistent social norm. Both adolescent girls’ and their mothers’ conformity to empirical and normative expectations was held in place by anticipated sanctions and high sensitivity to the sanctions. Being successful at school and having supportive schoolteachers were the most common conditions/exceptions for avoiding early marriage without facing sanctions in this study context.

The use of locally developed and tested vignettes to understand social norms was the main strength of the study. The use of vignettes with locally relevant stories was very helpful for initiating discussion on this very sensitive issue, early marriage. Normally, engaging all participants on a sensitive matter in an FGD is challenging. On the other hand, FGDs are commonly used to study collective behaviour/norms. The use of the vignettes helped to reduce sensitivity to the issues by introducing a common story for the participants [19]. The data sources were triangulated to deepen our understanding and interpret the findings considering different perspectives. The researchers also had several opportunities to have a prolonged stay in the field, which helped them to conduct a more open dialogue on the matter [24]. This study is believed to have shed light on the social norms related to early marriage in the West Hararghe context; social norms are deeply entrenched in local culture and, as such, a universal understanding is not helpful in designing local interventions [25]. As deeply entrenched phenomena, social norms evolve slowly and changes may not be uniform across the country [26].

In this study, early marriage was a conditional preference because adolescent girls and their parents conform to the local social norms. Conformity to social norms is dependent on the deeds and reactions of other members of the society [9]. Conformity to marriage social norms happens even when local law prohibits early marriage; in the Ethiopian context, marriage before 18 years is legally prohibited [27]. Parents cover up early marriages by not referring to the ceremony as a wedding; rather, they claim the occasion is for another locally celebrated event [13]. The girl and her partner may also run away to be together without parental consent; often, they come back to their village after some time, which is a self-declaration of marriage. Since girls are believed to be unmarriageable once they lose their virginity [28], parents would accept such a ‘run-away’ marriage.

Adolescent girls’ marriage as a conditional preference aligns with Bicchieri’s description of empirical and normative expectations [29]. This study adds to the existing literature, in particular by indicating how social norms play an important role in perpetuating girls’ early marriage by persuading young girls to willingly accept marriage proposals when a suitor shows up. Young adolescent marriage decisions are highly affected by their peers and other influential members of the community [30] who sustained social norms by injecting sanctions for deviant practices [26].

Sanctions such as insults, discrimination, and defamation play crucial roles in enforcing locally prevailing early marriage social norms. Fear of isolation and the severity of consequences often force young adolescent girls to abide by societal norms [9, 31]. The strong human desire to belong to a group/community also enforces social norms [32]. Zasu explained sanctions to be of two types: formal sanctions for breaking laws or formal agreements and informal sanctions for defying informal unwritten societal rules such as social norms [33]. Although informal sanctions are not written, people in the community acknowledge and enforce them. Informal sanctions are collectively imposed and hard to ignore; thus, societies use them to sustain local practices [34]. Therefore, people in the community are inclined to conform with practices most acceptable to those regarded as influential to them and the society they live in. Peers and other reference groups hold key roles in sustaining social sanctions [35, 36]. Psychological and physical sanctions such as insults, defamation, and abduction are used to enforce social norms [37]. These findings around sanctions are consistent with other studies conducted in different settings and countries where early marriage practices are highly prevalent [31, 38]. Strong sanctions for refusing marriage were reported previously in similar contexts [5, 39, 40]. Young adolescent girls in rural Ethiopia lack agency and sanctions may have a stronger effect on their lives [41].

This study identified certain conditions (exceptions) that would make deviating from the early marriage social norm acceptable. We found that girls’ performing well in school and having supportive teachers who are capable agents are important conditions to avoid early marriage. Teachers are often well respected in rural Ethiopian communities and can play important roles in addressing unfavourable social norms in a way that benefits young adolescent girls [42]. Consistent with our study, strong educational/school support was identified by previous studies as a prominent remedy for avoiding early marriage [42, 43]. Schools are important platforms for early marriage alleviation because they promise an alternative for the girls’ future through success and independence [1]. Early marriage versus education competence for girls’ youth has been an issue for a long time in many settings [44]. Girls’ strong determination for education may emanate from observing better opportunities educated women are experiencing in their context [45]. Girls’ determination to pursue their education, if coupled with strong school performance and supportive school administration, is critical for avoiding early marriage [42]. The platform for intervention may vary from context to context; there could be platforms other than schools that can be used to stage interventions against early marriage [25, 46].

Early marriage is a physical and mental health burden on girls and lingers throughout their lives [47]. It increases susceptibility to gender-based and intimate partner violence as it diminishes the chance for better education and independence [48]. Addressing the issue of early marriage is one of the components of Goal 5 of the Sustainable Development Goals (SDGs). Target 5.3, in particular, aims to eliminate all harmful practices such as child, early, and forced marriage and female genital mutilation [43]. The *Girls Not Brides* initiative also indicated that eight of the 17 SDGs might not be achieved without significant progress to end early marriage [49]. Ethiopia has signed this United Nations’ global development goal to end early marriage by 2030. Despite all these commitments, early marriage continues to ruin the lives of millions of girls in low-income countries [50]. Thus, studies such as ours are important for strengthening early marriage intervention efforts by depicting local social norms that are critical to making interventions context-relevant [25, 50].

The process of developing and actually using the vignettes was time-consuming. However, we strongly believe the process was very critical to understanding locally important issues in addressing early marriage, as social norms, sanctions, sensitivity to sanctions, and conditions can vary greatly by context. Apart from facilitating the researchers’ prolonged engagement with the local community, the process also allowed for engaging local stakeholders. Due to the sensitivity of the issue and the conservative nature of rural communities, some of the participants of the study may have been reluctant to freely and thoroughly share their views in focus group discussions. However, we opted for FGDs, as vignettes work best in group settings.

## Conclusion

In this study context, social norms strongly encourage early marriage and are mainly perpetuated by peers of adolescent girls and influential adults. A strong determination to continue education on the part of girls, strong school performance, and supportive schoolteachers are important conditions for circumventing social norms on early marriage. As social norms evolve slowly, we recommend periodic assessment in order to develop locally appropriate interventions against early marriage.

## Supporting information

S1 Appendix Vignette based study tools. (PDF)

S1 File Focus group discussion transcripts. (ZIP)
