# Supplementary material for: Adolescent girls trapped in early marriage social norm in rural Ethiopia: A vignette-based qualitative exploration
Source: PLoS One. 2022 Feb 17;17(2):e0263987. doi: 10.1371/journal.pone.0263987 (PMC8853576; doi:10.1371/journal.pone.0263987)
Supplement: S1 Appendix — (PDF) [file pone.0263987.s001.pdf]

Study tools: Vignette based Focused Group Discussion Guides

A. **Study tool for adolescent (married girls, unmarried girls and boys)**

I will tell you a story of a girl I named Rehima (that is not her actual name) living in this district. I would like you to listen to the story carefully and discuss the questions that follow.

Rehima is a 16-year-old student who lives with her parents. She attends school and helps her mother with household chores. One day Hindiya, Rehima's cousin comes over to visit Rehima's family. They are about the same age. Hindiya announces that she is engaged and getting married in a month's time. She also strongly suggests to Rehima that she should also marry soon as she is getting old for marriage. Hindiya reveals that she also knows someone from their village who is interested in marrying Rehima.

a. What would most adolescent girls in Rehima's position do in this situation?
b. What would Hindiya and most other girls expect Rehima to do in this situation?

But Rehima doesn't want to marry young. She announces that she does not want marry at this age.

c. What would Hindiya and most other girls say about Rehima's decision?
d. Would the opinions and reactions of her peers make Rehima change her mind about refusing the marriage?
e. Are there any circumstances where it would be considered more or less acceptable for Rehima not to get married at her age?

B. Study tool for parents (mothers and fathers)

I *will tell you a story of a Mother named Merima and her daughter Asha. Let's pretend they are from this Village. I don't want you to think about a real Merima and Asha who lives here. We could have chosen other names, but for now let's stick to those. I would like you to listen to the story carefully and discuss the questions that follow.*

Merima is a mother of five. She has three daughters, of whom the eldest [age17] had moved back with her after divorcing her husband of 1 year. They all live in the same compound, including her sons and their wives. One day when all of them plus some mothers in neighborhood were sitting at a coffee ceremony in their compound, an intermediary (*delala*) comes to their house and tells them that Merima's younger daughter Asha [age 15] is being sought by a young man for marriage. Asha firmly announces that she is not interested to marry any time soon.

a. What would most other mothers like Merima do in this situation?
b. What would most other mothers advise Merima to do regarding her daughter's refusal to the proposal?

Let's return to the story. Merima listens to Asha and tells her neighbors that she respects her daughter's decision to delay marriage until she's older.

c. What would most other mothers say about Merima in this situation?
    - What would other people such as the *delala* say about Merima?
d. Would the opinions and reactions of the other mothers make Merima change her mind about her daughter's marriage?
    - Would their reactions have an effect on her?
        - If so, what would be the effect?
        - If no effect, what makes you think that way?
e. Are there any circumstances where it would be considered more or less acceptable for Asha not to get married at her age?
