# Supplementary material for: Adolescent girls trapped in early marriage social norm in rural Ethiopia: A vignette-based qualitative exploration
Source: PLoS One. 2022 Feb 17;17(2):e0263987. doi: 10.1371/journal.pone.0263987 (PMC8853576; doi:10.1371/journal.pone.0263987)
Supplement: S1 File — (ZIP) [file pone.0263987.s002.zip › PDF FGD data/FGD 01_UAG_D3.pdf]

Unmarried adolescent Girl_District 3

**Discussants background information**		
Name initial	Age	Education (Grade)
MA	15	7
IA	15	7
NMA	14	7
HS	15	7
NM	14	7
BH	14	8
RA	13	8
MM	15	8

Marriage vignette

F: Rehima 16 years old a student who live with her parents, she attends school and helps mother with household chores. one day Hidiya, Rehima's cousin came over to visit Rehima's family. They are about the same age, Hindiya announces that she is engaged and getting married in a month's time. She also strongly suggests to Rehima that should also marry soon as she getting old for marriage. she reveals she also know that there is someone from their village who is interested in marrying rehima.

F: What would most adolescent girls in rehima's potion do in this situation?

R1. I will tell her as she can get marriage but as I am learning and help my family, because my parent also allow me to learn.

Also age is not passed to marriage. So I have to learn fist.

R8. As our village culture, girls when somebody want to get marriage her peers, immediately wants to get marriage because she fears when her peers get marriage, she may feel as her age gone and as she will not get husband.

If I were her, I would decide to get married if proposed to. … Education is for making a living; so is marriage. I will drop (out of school) and marry. When she (Hindiya) tells me that somebody

wants me for marriage, and that she will get marriage soon, I will also deicide to get married immediately.

R3. In between our discussion, when she tells me that my age is passing for marriage and when she tells me she will marry soon, I will accept her advice because I fear my age to not pass for marriage. If my age passed to marriage, I know nobody will marry me, so I have to accept her advice and deiced to marry that guy (shy laugh) this is our culture of marriage.

F: What would Hindiya and most other girls expect relying to do in this situation?

R1. They will expect her as she will get married. Like as she will discontinue her education, as she will get marriage by introducing the guy that he wants to marry her.

Because they expect her as to get marry rather than learning at that age.

R2. As she accepts her advice and automatically gets marriage and as she have to live with that guy by marriage and stop education.

R8. As her friend will introduce her with that guy to marry her.

F: But Rehima doesn't want to marry young. she announces that she does not want marry at this age.

F: What would Hindiya and most other girls say about Rehima's decision?

R7. As education is useless and should stop learning to get marriage.

R6. She will lose her interest to learn when her peers advise her like this.

R5. They will say that everything will be for living so it is better getting marriage rather than learning because if she is not get marriage at this age, later as she will be ugly and nobody want to get her for marriage.

So it is better get marriage now by this age. The peers also try exhaustively to change her learning idea and get marriage.

R4. The peers also think why we learn? She will get marriage so we have to stop education and get married.

R5. The community tries to as she will hate education and decide to get marriage.

R5. If I were her, I would try to continue education and after that I will think about marriage because getting marriage at any age is possible have interest to learn, I will not accept her idea.

R3. I will leave her as she will continue her education.

F: Would the opinion and reaction of their peers make Rehima change her mind about refusing the marriage?

R2. If she will be despair for learning due to her peers, she will change her idea decide to get marriage.

R6. Yes, like peers may tell her as they will have offspring for memories, if they die but as she will not have offspring for memories if she dies.

So due to this idea they can change her decisions.

R2. She will change her idea for she will not have offspring unless she will get marriage now.

F: Are there any circumstance where it would be more considered or less acceptable for Rehima not to get marriage at her age?

R2. If she could convince them as learning is important for human life, Like if she educate up to the end as she will employed, and if she will not educate as she will daily fetch water by donkey, and as education rather than marriage make human wealthy.

R8. If we think as marriage is useless and education is better than everything and deicide to learn.

R6. If those influencing peers were student, she can easily convince them as she has to continue her education rather than getting marriage at this age.

But as her peers are many in number as you now many people idea can win them what so ever one alone specks.

So peers can influence her continue education and get married.
