# Supplementary material for: Adolescent girls trapped in early marriage social norm in rural Ethiopia: A vignette-based qualitative exploration
Source: PLoS One. 2022 Feb 17;17(2):e0263987. doi: 10.1371/journal.pone.0263987 (PMC8853576; doi:10.1371/journal.pone.0263987)
Supplement: S1 File — (ZIP) [file pone.0263987.s002.zip › PDF FGD data/FGD 02_MAG_D3.pdf]

Married adolescent Girl_District 3

Discussant background information		
Name Initial	Education (Grade)	Age
MA	04	17
SY	04	15
MA	04	15
HA	illiterate	15
MA	01	15
MN	03	17
CHA	illiterate	17

Marriage Vignette

Rehima is a 16-year-old student who lives with her parents. She attends school and helps her mother with household chores. One day Hindiya, Rehima's cousin comes over to visit Rehima's family. They are about the same age. Hindiya announces that she is engaged and getting married in a month's time. She also strongly suggests to Rehima that she should also marry soon as she is getting old for marriage. Hindiya reveals that she also knows someone from their village who is interested in marrying Rehima.

F: What would most adolescent girls in Rehima's position do in this situation?

P1: What she can say was if she has an interest of education I have to continue my school and I don't want to marry. The other girls said she has to continue education once she reaches this level.

P2: The other girls said please continued your education and don't marry.

P7: you know people are diffident. Some said marry and the other said continue your education.

P8: The other girls like her said that marry and the education were useless.

P5: If she wants to marry, she can marry.

P3: If she has fascinating of marry, she can marry.

P4: The other people said marry was not important, so please continue your education. They don't encourage her to marry.

P1: The other girls expect as she married. The expected as she not learned education. The other girls expected as she married and not toward education. They expected as she do what they order them.

P6: The other girls expected as she do situation they are living. They expected as she drop out school. .

P7: The other person encourages her to education

P6: The other girls expected as she to become like them. They expected as she diverted her attention towards marry. We also abandon education engaged and getting married. She should also have engaged and getting married like us.

P1: Once she engaged they advised her to getting married.

F: What would Hindiya and most other girls say about Rehima's decision?

P1: The other girls they encourage her to married and told you are at the age of marriage. They prevent her as she not learned education. If she was wanted to learn then by saying this divert the attention of her. They said you have reached for marry and stop education. They can say what the education can do for you, you are not young and you have to married.

P6: They can say what the education can do for you and advice for marry.

P4: We also leave education, what the education can do for you. They said married. They think or searches the way she leave school.

P7: if she wants to learn, she can refuse what they said and can learn. They said please leave education then engaged to marry. They said we will give for you.

P5: they said please leave education and they encourage her to marry. They said if you have an interest of marry they encourage her to married.

F: How would that make Rehima feel?

P1: if she has an interest of education her idea was not changed. The family of her if learns that she wants to go school, they encourage her to education. If she doesn't want marry, they can't do nothing. We are enforcing her to marry but she doesn't want to marry then we should have to leave from her such issue. They said she has lean for us and stop such issue from her.

P4: if she has an interest of education it is difficult to change.

P3: The reactions and opinions of her peer they can her minds refusing marriage. The peers of her don't want education because the engaged and getting married and they can change her to marriage. The peers always talk to her about marriage so many times about her then divert her minds. They were tried to steal her minds by saying the education can't do nothing for you and where the educated person go or reach then make as she married.

P6: the opinion of her peers can change. The peers of her they don't have an interest of education. As a result of this they can changed her minds. They tell for her where the educated person can't reach.

P7: They tell education was useless and they tell marriage was better than education.

F: Are there any circumstances where it would be considered more or less acceptable for Rehima not to get married at her age?

P6: If she was refused to marry, it is not acceptable in the society because according to community in our area they support marriage than education.

P1: my idea was what she already said.

P3: it is not acceptable refused to marry. They said marriage was better than education. If you learned education you can't reach any place.

P5:It is not acceptable refused to marry at the family of her because during the ancient they were married each like this situation. They are illiterate because of they encourage her to marry. If married she can't do. They said females once married they can't go anyplace. They said education was not important, the cant do nothing for the person. If you learned education, you can't reach anywhere.

P1: If one girl engaged in marriage, she was decided by herself. So there is no reaction or influence from the person. If she was married at young age and married by her interests then they can give her. Tis means the family doesn't have another option to agree with her.
