# Supplementary material for: Adolescent girls trapped in early marriage social norm in rural Ethiopia: A vignette-based qualitative exploration
Source: PLoS One. 2022 Feb 17;17(2):e0263987. doi: 10.1371/journal.pone.0263987 (PMC8853576; doi:10.1371/journal.pone.0263987)
Supplement: S1 File — (ZIP) [file pone.0263987.s002.zip › PDF FGD data/FGD 03_MOAG_D3.pdf]

Mothers of adolescent girl/s_ District 3

Discussants background information			
S.No	Name initial	Education	Age
1.	AK	Illiterate	30
2.	RM	Illiterate	40
3.	HA	Illiterate	40
4.	HH	Illiterate	40
5.	SD	Illiterate	45
6.	SA	Illiterate	35
7.	AY	Illiterate	40
8.	AA	Illiterate	40

Marriage Vignette

Merima is a mother of five. She has three daughters, of whom the eldest [age17] had moved back with her after divorcing her husband of 1 year. They all live in the same compound, including her sons and their wives. One day when all of them plus some mothers in neighborhood were sitting at a coffee ceremony in their compound, an intermediary (delala) comes to their house and tells them that Merima's younger daughter Asha [age 15] is being sought by a young man for marriage. Asha firmly announces that she is not interested to marry any time soon.

F: What would most other mothers like Merima do in this situation?

P1: (One discussant said); do you mean her neighbors?

F: Yes,

P2: As to my idea; The elder one already divorced, she is not in marriage any more. First you had allowed marriage for the person who is not able to manage her, he has nothing. You do not need to commit same mistake, you have to follow you follow your little daughter Asha, look after

her; they are going to take her, she may be back to home after marriage." This is what other mothers say.

(Assertively replied); other mothers say; *"do not be barmy or foolish, people are going to cheat you again, your elder daughter already failed, she has divorced, if allow the little one she may be back with children which is worries you have to decide carefully protect her", other mother actually say or do this.*

F: (What about you (gave chance for shy discussant?)

P3: (she ask to repeat the question and the question repeated); the other mother from neighbor may say, *"convince your daughter for marriage; she has to marry",* there are also mother who do not support marriage of Asha, but as to me I do not allow this marriage because Asha is 15 years of age, she is bellow ideal age of marriage.

F: Please we want to know the actual practice and norms of marriage in this locality?

P4: any other mother like Ashas' mother advise her, we also do same thing, we cajole Ashas mother, to advice Asha for marriage.

Ashas' age is not right age of marriage, people are trying to convince her, and they are going to affect Ashas' education ... (other discussants make noise and terminate her), most hear sound were "age no...no problem..." one dominant voice heard was; "we allow the marriage..."

"...Asha may not be student, nothing mentioned about education..."

(the chance is given for one discussant and she said), as to my idea, Marimas daughter is not willing to marry, the first daughter tasted marriage and divorced, this is also obvious, what we usually experienced, but now she do not need to allow the current marriage

Mothers advice Asha's mother to allow marriage; *"this is good chance the boy has also properties"* saying this some mother made the marriage to happen. Sometime mothers wish to see the marriage of their daughters.

F: Any more please, tell us the most common practice of the community, the real norms of the community on adolescent marriage.

P5: Some mothers consult on the issue, she tries to cajole the lady and made here to get married. From the story of Asha and her mother, when such news related to marriage heard in the family, this indicates that some body is following her, she is under concern/interest of somebody. Due to this what we can decide about Asha is, she has to get married, we consult on it saying; *“the parents of this boy is rich, they have good family background, Asha got good chance, she has to marry”*, this is what most of us decide about Asha.

F: What would most other mothers advise Merima to do regarding her daughter’s refusal to the proposal?

P6: (Replied loudly), the advice of most mother is; “marriage is chance, it is good fortune try to advice your daughter smoothly, convince her to accept the marriage, she has to marry”

Some mothers also advice saying; “keep your daughter from the person walking around her, probably that person the one who is willing to marry her”

F: Based on the norms of marriage in this community, please tell me what most mothers actually advice Marima to do?

P7: Since the age of the daughter is little, she is under age, she do not need to get married (smile...)

F: Frankly speaking is Asha, 15years old is under age of marriage, if she is in this locality?

P8: culturally we do not have specified age of marriage; there is no predetermined age of marriage. If the two adolescents, boy and girl agree and love each other, we can’t forbid them because this is the trends and culture of our foremothers and fathers. If the girl is willing and fortune happens to occur, we immediately allow the marriage. Sometimes intermediaries enforce girls and convince them to marry someone; it is responsibility of parents to save their daughter from such person. Otherwise marriage and death is same; both are unavoidable events in a life.

F: What else please tell us your idea, in relation to the age of marriage?

P7: age...in our culture age... Marriage...one person dominates and replied, in the culture of this community age of marriage is, 16 years, 15 years. Whether the age of marriage is determined or not, some girls married at the age of 20. That is it; people do not refuse to accept marriage based on the little age of daughter.

F: Any more please?

P5: Uhuuu!!! Said all discussant and quite. We advice to allow marriage at any age *(She assertively replied*) The neighbors' advice is mostly, influencing the mother to allow the marriage. Based on the norms of our community no one encourage the mother to respect the interest of her daughter. Almost all mothers, say; *"it is good to allow this marriage"*

P6: whatever the age of the girl, whether she is little girl or not, we encourage her and say; *"allow this marriage"*. To tell you truth of this community, no mother refuse marriage, even if the girl do now want to marry, if she strongly refuse we try to persuade her as much as possible. Sometimes the girls' may agree to get married, but mothers may refuse. In such situation, we influence the refusal of the mother, "you are refusing for no value, keep quiet, this is the interest of the daughter and culture of our community, you perceive your daughter is not old enough to get married, but she is saying I love him, she may leave the house one day you have to allow this marriage" saying this, we change the mind of the mother to allow marriage.

Convincing the mother is expected from neighbors; so we persuade her saying; "stop refusal and simply celebrate your chat/khat, chew your chat/khat with elder people, what if the girl go with the boy, are you going to take her back? This is impossible, this sis good fortune. Do not affect such fortune of the girl", such thought can easily persuade not only mother, but also fathers. Mother never accept marriage refusal of girls.

P3: Based on the previous culture of the community marriage could be at 18years; but currently girls are fast for marriage, due to this if the she reaches 12 years of age and somebody ask for marriage we immediately allow marriage.

F: Let’s return to the story. Merima listens to Asha and tells her neighbors that she respects her daughter’s decision to delay marriage until she’s older.

What would most other mothers say about Merima in this situation?

P1: What people say about this is; *“allow this marriage, convince your daughter, even if Marima refuse, other mothers persuade her, they hold her down and allow marriage”* this is what we have been doing for a longer time, whether mother or father feel and or cry, people have been .

P3: there is the time your daughter decide and you do not have such chance of involvement, now you have got the chance to give decision at your home. If something happen to your daughter and you ask for support from neighbors, neighbors can do everything perceived is better for the daughter. This is not ‘jala deemaa’, people are looking for your willingness and respect for you”, when we talk to Marima like this she can be persuaded and decide to allow marriage. We (peer mothers) try to convince the mother (of the proposed-to girl), saying, ‘if you refuse now, you may be humiliated if your daughter elopes with the boy, which will be a big shame to you and your family.’ This is the culture hereThey all say, “yes” in one voice.

F: What would other people such as the *delala* say about Merima?

P5: *(Aggressively replied with loud voice),* if Marima is strong enough o keep her promise and refuse not to give her daughter for marriage, she can actually do it. it not only mother, neighbors and people in the community shout on the mother, confuse her and made her to allow marriage. If people support the idea of mother and Asha, than pushing for marriage it is possible to save her from marriage.

P4: such culture of allowing marriage at any age any time, is strengthened by the community, by people, people talk this and that, the marriage is allowed in minute.

F: What do Marema feel or do from the reaction of other people and Delala?

P2: She gets weakened; she can’t keep her stand or idea, since all people influence her. When many people influence anybody, that person becomes weak, same thing happened to the

mother. Due to this she feel angry, get annoyed. No mothers simply allow marriage without the influence of people. Mother doesn't feel happy for allowing marriage, because everything is from the influence of the other people; *"the daughter is not your alone, she is ours too we can decide on her."* What can she do after all? Does she have anything to do? She gets mad from irritation cry and can't do anything. Even though she gets crazy; when she allows marriage, another bad problem may happen if the marriage of her daughter fails. If Marima's daughter divorced and go back to her mother's house, that will be the time Momina strongly annoyed and regret for allowing marriage with the influence of her neighbors. She mat suffered a lot, because she is going to live with two divorced daughters. *"I could have to keep my promise, I could not allow this marriage"*

P3 such type of bad feeling and problem, if the marriage is successful it is happiness, happiness no sadness at all.

When daughters married, mothers feel happy. The happiness of girl is happiness for mothers also. Especially when marriage is successful, the happiness is endless.

F: Would their reactions have an effect on her?

P4: What effect is greater that enforcing mother to give her daughter for marriage? She is enforced, influenced by neighbors while she was refusing. No effect more than this.

If the mother strongly refuse her daughters marriage, people warn her saying; *"you are cursing your daughter, attracting bad fortune, attracting curse to your daughter"* due to this the mother fear such thought.

F: Are there any circumstances where it would be considered more or less acceptable for Asha not to get married at her age?

P6: Once the fortune happens for Asha, she could have to marry.

If Asha hate the boy and doesn't want to marry him, she may be accepted. This can only be accepted if her parents support her idea.

(She assertively replied, with motivation), when human being is born, whether that person is boy or female marriage is a must at right time, at right age. Girls must look for husband and marry something could be dome for boys also!!! (replied loudly)

P7: Marriage is unavoidable it is a must!!!

At right time no one can be out of married, everybody wait right time of marriage that is it.

Marriage the gift of God, it is only God that can avoid marriage. Marriage can be absent due to death only, when it is right time marriage is a must, nothing can make it impossible.

F: Concerning the discussion we had till now; do you have any additional idea?

P4: Yes, (one discussant said); marriage issue is becoming expensive. The marriage of current time is not as it was before. Currently marriage is possible only for rich people, it is becoming impossible for poor people who can't pay money for daughter's parent. Poor people who do not have 10,000 birr can't marry, no way. This is culture, the challenge is bigger among parents of the boy.

F: Uh...can we say marriage doesn't exist in the current time?

P4: Hay!!! Nothing make it impossible unless death.

(They all laugh.......). If she is on education, probably she may stay longer than ideal age of marriage. Adolescent boys, agree with the girls and go together, they actually doesn't have a single coin, but they decide themselves.
