# Supplementary material for: Adolescent girls trapped in early marriage social norm in rural Ethiopia: A vignette-based qualitative exploration
Source: PLoS One. 2022 Feb 17;17(2):e0263987. doi: 10.1371/journal.pone.0263987 (PMC8853576; doi:10.1371/journal.pone.0263987)
Supplement: S1 File — (ZIP) [file pone.0263987.s002.zip › PDF FGD data/FGD 04_UAB_D3.pdf]

Unmarried Adolescent boys_District 3

Discussants background information		
Respondent Name Initial	Age	Education
**P1. SA**	15	8
**P2. EA**	16	7
**P3. AA**	15	7
**P4. SI**	15	7
**P5. RS**	16	8
**P6. AS**	17	7
**P7. SA**	15	8
**P8. SS**	18	8

Marriage Vignette

Rehima is a 16 year old student who lives with her parents. She attends school and helps her mother with household chores. One day Hindiya, Rehima's cousin came over to visit Rehima's family. They are about the same age. Hindiya announces that she is engaged and getting married in a month's time. She also strongly suggests to Rehima that she should also marry soon as she is getting old for marriage. She reveals she also knows that there is someone from their village who is interested in marrying Rehima.

F: What would most adolescent girls in Rehima's position do in this situation?

P8. They decide to merry, when they aged by following each other they also fall in such decision.

P7. She allows such idea, my fried have been married am also have to.

P6. She does not tarn over, it so she marries, so she accepts the idea.

P5. She does not turn back so she marries their understanding is that one and her family also influence her by don't lose the chance. If she married soon, she looked as loved one. And if she married lat she looked like as love less one.

P4. She fulfills what she has commanded also if she is on education, she break the education and back for marry. It looks like unavailable chance if she loses one.

P3. They also support her, and she also like to marry.

P2. She accepts by saying okay.

P1. She accepts what her friend said to her. If she loses a chance, it seems to her as unavailable chance.

F: What would Hindiya and most other girls expect Rehima to do in this situation?

P1. .what they say she accept as, because it's obvious idea.

P2.what they say she accepts as, because it's obvious idea.

They act as their friend and marry together.

In other case conditioner is uneducated family. They intermediated by their friends and block each other and forward their faces to marry, and the man who went her only giving money to intermediate. If he lose what he need, he drop her after make her life in difficult.

P5. There is a sign to identify whither she is virgin or not so that. There is a word; if a girl loses virginity before married, her ear will be cute. So that the female keep herself before marry.

P8. She has been made to take her friend idea if she disobeys them; they try to influence her by thinking over it.

Sanction

P5. She gets the attitude of as she had a great mistake by both community and her friends. It looks like as she loses unrecovered chance.

She will not get boyfriend, she do not think what other do, they say she may also have another problem and if she lose the chance they think; she may have the problem of healthy, and knowledge.

P6. She loses acceptance, she may also get the attitude like she is abnormal. Averagely they marry at 15 and 16 year.

P4. They marry under 15 years. If she do not do she will be disrespected, she will be called unmarried left at her age and become who do not out of home. After that any one do not need her.

Who insult her some time is friends and much of the time them.

P8. Her friend insults her, among the family her mother also insults her more if she makes fail to marry.

So she does not like the word from them. As a word back and chat earn from her mother (she called as matured banana one). If the chat do not harvested at a time, it's lose. So they look like chat.

Sensitivity to sanction

P8. He can change her decision; also her girl friend can also influence her decision.

They think as that one is good and they all of them compromise on that idea.

F: But Rehima doesn’t want to marry young. She announces that she does not want marry at this age.

F: What would Hindiya and most other girls say about Rehima’s decision?

P5. She gets the attitude of as she had a great mistake by both community and her friends. It looks like as she loses unrecovered chance.

She will not get boyfriend, she do not think what other do, they say she may also have another problem and if she lose the chance they think; she may have the problem of healthy, and knowledge.

P6. She loses acceptance, she may also get the attitude like she is abnormal.

Averagely they marry at 15 and 16 years.

P4. They marry under 15 years. If she do not do she will be disrespected, she will be called unmarred left at her age and become who do not out of home. After that any one do not need her.

Who insult her some time is friends and much of the time them.

P8. Her friend insults her, among the family her mother also insults her more if she makes fail to marry.

So she does not like the word from them. As a word back and chat earn from her mother (she called as matured banana one). If the chat does not harvested at a time, it’s lose. So they look like chat.

F: Would the opinions and reactions of her peers make Rehima change her mind about refusing the marriage

P8. He can change her decision; also her girlfriend can also influence her decision.

They think as that one is good and they all of them compromise on that idea.

F: Are there any circumstances where it would be considered more or less acceptable for Rehima not to get married at her age?

P8. There is no possible condition.

P1. She have no solution, she is let due to do not get chance.
