# Supplementary material for: Adolescent girls trapped in early marriage social norm in rural Ethiopia: A vignette-based qualitative exploration
Source: PLoS One. 2022 Feb 17;17(2):e0263987. doi: 10.1371/journal.pone.0263987 (PMC8853576; doi:10.1371/journal.pone.0263987)
Supplement: S1 File — (ZIP) [file pone.0263987.s002.zip › PDF FGD data/FGD 05_FAG_D3.pdf]

Fathers of adolescent girl/s_ District 3

**Discussants background information**		
Name Initial	Age	Education (Grade)
MA	60	Uneducated
AA	50	Uneducated
YA	70	Grade 3
MS	49	Grade 5
AA	75	Uneducated
LF	35	Grade 3
HB	32	Uneducated
AA	66	Uneducated

F: Okay, now I read another history for you and we will discuss on it as the former one.

Marema is a mother of five children, where three of them are females. Among the three girls, the oldest one married at 17 years old. However, after one year she return to her mother's home as she divorced. Then all of them started to live together again. As such, one day while they are on the coffee ceremony with their neighbor; one person (intermediary) come to their home asked if they want to marry Aisha who is 15 years old to a given guy. However, Aisha told them not to marry at this age because she is underage.

2(a). According to this locality other mothers like that of Marema will do what if they come across this issue? If the girl refused to marry as per the request and her younger sister also divorced, so what other mothers do?

NT: If she refused what they do her?

Participant 1:

According to our local area, as far as she is requested to marry though her older sister is still; we advise to agree for the marriage.

Participant 2: My sister moved back because she don't know how to handle her family but I can handle so I will marry

What if the girl does not agree with her mother decision, and what other mother will do here?

Others also enforce her for marriage.

Let we discuss more on this issue/ any more explanation?

Participant 3: According to the locality, others also manipulate the girl to marry because there are some sorts of facilities like *barcaa* and tea in response to the marriage deal. Again if the girls agree with the intermediary they can marry to whom they like, and finally announce for their families even.

Participant 4: According to our area, we don't agree for underage marriage because legal age for marriage is 18 years old.

Let we discuss more on what is happening in this locality rather than what legally prescribed.

Participant 5: Here what is practiced usually even though I don't allow her she didn't ask me, as our community there is a khat chewing day in their rest day in that day children hold each other we can't protect them.

F: Is the girl allowed to go to the khat chewing day?

Participant 6: Even though the girl refused to agree with the marriage deal, there are other individuals who have more experience on the issue to regulate. Weather friend or neighbors negotiate and make her marry to get profit as a broker.

What if she refused again?

Participant:

If he is brave, lobbies more with more other three individuals and they manipulate her.

What could be the final decision, if she refused more?

Participant 7: If she refused at the end, then they quit the deal and the man search for another girl who may agree with the deal.

Is there anything that faces the girl who refused to marry?

Participant 8: The people may hate her by saying that she would not accept advice from her friends and families.

F: What will happen to her in the long run?

Participant 1: Nothing, but at the time she want she marry the one who brings her chance because no one left from marriage.

After she become old can she get husband?

Participant3: Once if she left her chance when she been wanted so her importance become very less

F: Let we say that, she refused the intermediary's deal then what other mothers like Marema will do on this case?

Participant 6: There is nothing exceptional they can do as far as she refused.

F: Doesn't she get any husband then?

Participant 6: No, she may find any other.

F: Is there anyone who ask her since she refused to do so before?

Participant 4: No one exists.

F: What will be done on her to be hated by others?

Participant 5: Others assume that she will also refuse them as the prior one, but if there is any influence on her she will go to another place to lead her career. In the case she refused while neighbors cooperate against her she leaves the place.

Ok now let us continue the second discussion

2(b). What advice can other mothers give for Marema to regulate the refusal of her daughter for marriage?

Participant 5: The only thing they can do is to assist Marema in the deal to convince her daughter.

For example, they tell her to influence her daughter for the marriage but if she refused they told her to let her as she like.

What shall they say, when they advise them?

Participant 2: They say to her mother you should have to give your child to the boy wants her, if she refused leave her us she want

Participant 7: If she refused while her mother and cooperated neighbors' advise her, they advise her mother "let her undermine she's say" making her do heavy work and do not fulfill her things what she need like cloths and others. And after that, the daughter left the town or even abroad.

Why she goes to abroad?

Participant 2: As her chance was passed once, she has no option rather than to do so.

Is there any more idea?

Participant 1: Yes, as far as she respects her mother's decision she is no more regarded as a daughter.

Participant 3: Once they cooperate against her they advise her mother, as far as she not accept your say she is not your child.

F: Okay, let we return back to the previous history. After Marema heard the decision of Aisha and she decided to stay home until her age reach for marriage and told the same to others. So, here as Marema decided in such a way is there any challenge she may face?

F: This means as Marema decided and agreed with her daughter not to married and announce to the neighbors', what will the neighbors say here?

Participant 1: Some neighbors say as far as the 12 years old girl engaged for marriage, the 15 should have to do the same rather they blame her as if she countered her daughter's chance for marriage.

Is there anyone who engaged for marriage at 12 years old in this locality?

Participant 3: Yes, if she is beautiful enough even at eleven years old. Beautiful girls marry early here rather than attaining school. But if mother deny this chance other blame her for that.

F: Okay, let you continue.

Participant4: Here our neighbor 12 years old girl moved with someone then her family went there and bring her back, then elders advise her and put her somewhere else and they appointed for the guy took her as he going to marry after 2 years when she reach for marriage.

Here, if mother agreed with her daughter not to marry what can other mothers say to Marema?

Participant 4: Just they simply respect her decision by saying that her mother is responsible for making her chance passed.

Do they blame Marema's for her decision?

Participant 6: Yes, they consider as if Marema closed her daughter's chance. And they say the F:

F: Here, as mother agreed with her daughter individuals such as agents/intermediaries who get some advantage from the marriage deal will say what? Is there any influence on the mother?

Participant 7: No, nothing.

Okay, as the mother closed her daughters for marriage what will other mothers do? Does they influence the mother to change her idea?

Participant 8: They may express their concern but they can't influence more.

F: Do they put sanction on her? Additional idea?

Participant 1: We say to her if you want you can engage your daughter for marriage, no more influence.

F: Then, what will the mother do as all neighbors blame her for which she closed her daughter's chance? Does she change her decision?

Participant:

No she doesn't change her decision.

2(e). As Aisha refused to marry at this age, does her refusal acceptable in the society?

Participant 6: Now a days it is acceptable, but not in the previous time. Now, here right is respected and her voice need to be heard.

F: But what is done here in order to convince her?

Participant 5: As she said her age is not reached for marriage nothing more exist to convince her.

Does it is acceptable as to this community? As she is 15 years old and refused to marry, does this is acceptable in the community?

Participant 6: According to our locality there is no any girl who refused to marry. Even at nine or twelve she will marry.

F: Is there any in which her decision/refusal is accepted?

Participant:2: Yes.

F: If her age is 15 can she refuse to marry? If it is what reason her refusal is accepted?

Participant 2: If she is following her education her decision is accepted even though several marriage deals come from agents/ intermediaries. Again, we are happy if she refused the amount of money that come for marriage deal to continue her educational career.

F: Are all communities happy with this?

Participant 3: Yes, they are.

F: Do they say, she lost her chance?

Participant 4: Since she is following her education, she don't lost even she may get more chance.
