# Supplementary material for: Adolescent girls trapped in early marriage social norm in rural Ethiopia: A vignette-based qualitative exploration
Source: PLoS One. 2022 Feb 17;17(2):e0263987. doi: 10.1371/journal.pone.0263987 (PMC8853576; doi:10.1371/journal.pone.0263987)
Supplement: S1 File — (ZIP) [file pone.0263987.s002.zip › PDF FGD data/FGD 06_MAG_D2.pdf]

Married adolescent Girl_District 2

Discussants background information		
Name Initial	Education (Grade)	Age
FU	04	17
SK	Illiterate	17
AM	04	16
HJ	02	16
HM1	02	15
HA	02	16
HM	03	17
HM2	02	14

Marriage Vignette

I: Rehima is a 16 year old student who lives with her parents. She attends school and helps her mother with household chores. One day Hindiya, Rehima's cousin came over to visit Rehima's family. They are about the same age. Hindiya announces that she is engaged and getting married in a month's time. She also strongly suggests to Rehima that she should also marry soon as she is getting old for marriage. She reveals she also knows that there is someone from their village who is interested in marrying Rehima.

What would most adolescent girls in Rehima's position do in this situation?

HA: She was attending school, Hindiya tells for her that one person was looking you for marrying. She was in dilemma. She asked herself to marry, hindiya was married and I have to marry like her. She can marry like this manner.

AM: She was not with us. She came here to visit us. As my opinion she should stop marry and completed her education then after get job she should think about marry.

HJ: she was married. I think as to marry then I will marry.

FU: You know the people in rural has very small brain. They said she was married and I have to also marry by saying this it ends from the school.

SK: After she told for me I decided to marry.

HA: For example, I married while I was learning… I got married when I was in fourth grade and while I was still in school because most of my friends were getting married. ... I became alone. Then, I said to myself, why would I go to school alone? … That is how I got married.. Other her peers they expected as she learned but most of her peers already married they expect as she will be married. Another friend expects as she married.

I What would Hindiya and most other girls expect Rehima to do in this situation?

HJ: Hindiya and other person expect her to marry

HA: When we see as our area they said x was married why I am seat then I have to married also by saying most of the girls in our area like this manner. The girls in this area go to the dance house or the music house. When they go there they can feel different things. As result of this she was passed or went. She drops out her school then married.

HJ: the people said to her what the educations do for you please marry. The literate people have no place and the literate people who are sitting were huge in number. They said please keep quit and married. They said if learned you were no reach elsewhere.

AM: after studied you have no place to reach. So please marry.

HM: They called one of her friend name. They said she was educated but not reach elsewhere. Therefore, you have to marry. As result of this, she was diverting her attention to marriage.

HM1: For example, all of us who are here was married. She was married and got child. If we studied, the educated people have no place. The literate people who are unemployed was many. When we are talking this kind of information in village they we are influence each other to face the problem of marriage and finally we will end up with marriage.

HJ: they mentioned name of the person who has completed grade 10 then said he was completed the education and where he is reached. Please don't play with your age and encourage her to marry. This is the chance and please don't pass this opportunity then divert her attention to marriage. To change her idea they said this different kind of opinion.

HA: this chance not passed you. For example Mr. x was learned till this grade and he was here. Don't play to your time; "Khat and Girls were morning" by saying these words then demoralize her. Means if the khat was not sold at morning then khat has no market at the afternoon. This means your chance was passed; it passed you by saying this idea so many times. After that she was feeling and thinking about marry.

HM2: the market of "Girls and Khat" was once or at a time. Means once it passed you, you never return to get it. This means that if you were not sold the khat at the morning the market was passed during that time. Therefore, your chance was once. They said marry once it passed you can get it by saying this and convince her to marry. Thus, abduction and arranged love would be go with her.

HA: Breaker or cabsa and arranged married was here. For the first time he brings khat for discussion. The khat was opened. If they chew khat it happens for him. For the second day he comes and inserts the stick on the wall. The name of the tree was urgessa, which has leaf and green. Once that tree was inserted on the wall of house that girls will be given for that person. It is not culture to prevent that girl. After that they said Khat and girsl was at the morning then given for candidate. If that stick tree was dried on the wall of house she will not be married. Therefore, once that stick tree comes and inserted on the wall of house it can't be reply. The abduction or breaker was undertaking at early morning and evening during family chew khat. The friends of that person or boys were inserted that tree on the wall of house when no one see them.

HJ: I married by Cabsa or the stick of tree inserted on the wall. The friends of my husband come at the evening and inserted that stick on the wall of house. My father was not seen. When my father lookind around there is a stck of tree put on the wall. He considers that these young boys were come for his girls. After that he has no option except giving his child. The elders come and call the father of her they said girls and khat were at the morning, so let us give your child for him. The other is if one girl refused to marry and call her let us go house dance. After that she and the person who like to marry her will be left in secret place. After that you have stayed a lot of time with him. So , you have to marry him. They married each other. They tried to intimidate her by saying after they sit together this all time. It is shame to go another place. So you have to marry him.

HJ: if she was not go that dance house she will not marry or if she does not give face for cabsa or breaker she will not marry. The acceptable condition was if the parents and girls was agreed on her education or discussed each other on her education. For example, I know one girl who is learned grade 3 with me. She has no father but has mother. She wants to study. All the times when the people tried to take her girls by cabsa or breaker, she was not allowed give her. Even you put her in secret place and stayed with that boy she come and take her child. Therefore, the mother of that girl was not given her child or girls then by saying this they leave her. Therefore, the commitment of family or parents was decisive.
