# Supplementary material for: Adolescent girls trapped in early marriage social norm in rural Ethiopia: A vignette-based qualitative exploration
Source: PLoS One. 2022 Feb 17;17(2):e0263987. doi: 10.1371/journal.pone.0263987 (PMC8853576; doi:10.1371/journal.pone.0263987)
Supplement: S1 File — (ZIP) [file pone.0263987.s002.zip › PDF FGD data/FGD 07_UAB_D2.pdf]

Unmarried adolescent boys_District 2

Initials	Age	Education (Grade)
1. MA	15	7
2. AA	15	8
3. AY	15	8
4. MMA	15	8
5. HA	15	6
6. AAA	15	8
7. UH	16	8
8. MM	16	8

Marriage vignette

Rehima 16 years old a student who live with her parents, she attends school and helps mother with household chores one day Hidiya, Rehima's cousin came over to visit Rehima's family. They are about the same age; Hindiya announces that she is engaged and getting married in a month's time. She also strongly suggest to Rehima that should also marry soon as she getting old for marriage. she reveals she also know that there is someone from their village who is interested in marrying rehima.

F: What would most adolescent girls in rehima's potion do in this situation?

R8. If her age reaches for marriage and if she loves that guy she may decides to get marriage.

R4. Other also the same way to advice each other to get marriage.

R6. Yes it is right most girls want to get only that wedding day happiness only so immediately decide by accepting her peers advice.

Even if males also hear this like idea they will advice her to get marriage.

R2. In our community marriage has high acceptance and her interest to education doesn't matter whatsoever. So even if she is not interested to get married, people and peers make push to get married once somebody loves her.

R7. Peers especially those of going to marry and already married are highly influence on girls as to make get marriage.

Even girls also feel get marriage when her peers will go to marry.

R1. Especially these married girls are highly advice or influence on girls as they have to discontinue their education and get marriage. At that time she will immediately accept her idea and decide to get marriage.

R3. As in our culture if peers tell as she will going to marry other also immediately diced to get marriage.

R4. Now for she is student better if she not accepts her peers idea and continue her education.

R8. Incase if some other girls hear when two peers advice each other about marriage automatically decide to get marriage to have offspring for man is mortal. So every girls are highly sensitive to decide about marriage whenever they hear about somebody else marriage.

R5. Yes, most girls are foolish easily change their idea even she is student, not wants to continue if peer advice about marriage. They will say education is useless so it is better get marriage.

R8. Some peer also advice as they have to get marry unless as they age gone and left husband.

F: What would Hindiya and most other girls expect relying to do in this situation?

R3. As she will decide and get marriage.

R2. The some as she will accept her idea and get marriage.

R4. When everyone gets marriage all peers wants to get marriage.

F: But Rehima doesn’t want to marry young. She announces that as she does not want marry at this age. What would Hindiya and most other girls say about Rehima’s decision?

R3. If once she decide to do not get marriage it will be hard to other forced her to get marriage. So peers try to bring guy that he loves her or her to decide and as he will try to convince her to get marriage.

R5. If she will not be willing for marriage but she may in love with him so peers, try to convince her as she have get marriage him.

R7. If her decision was due to education, peers may advise her as learning is not use full for girls rather as have to get marry. If her decision was also due to age she will tell her as she is in the same age and not affected with age and as marriage would possible at this age.

R8;- Her friends will tirelessly try to influence her to accept the marriage, and if she refuses, they may even facilitate to get her abducted. This means for example when she goes to market, her friends make a deal with his friends and will go with her. When they reach open space, those friends of the guy will catch her forcedly and take her to his house, and as that time family send his father to her father. At that time her family as well as she herself will agree to marriage. Because once she spends the night at his house, she will be humiliated to return back with her parents. So the wedding will process. Due to this like method people are make girls as they discontinue their education.

F: Would the opinion and reaction of their peers make Rehima change her mind about refusing the marriage?

R1. Yes, they can make to change her decision: by telling her learning is meaningless and also convincing, as her age is enough to get marriage. And by telling with models like somebody learned and returned to marriage. Even here are some peoples murmuring as the government said that they don’t want any additional educated persons.

Due to this people are being despair for education, by picking this like idea they will convince her.

R8. Yes there are some girls example reached up grade ten and discontinue or failed may tell her as learning is useless only wasting time and even government not wants educated person.

At that time she will change her attitude from learning to get marriage.

R5. By advise her as she hate education and want to get marriage. Rather telling as she can get wealth if get marriage.

F: Are there any circumstance where it would be more considered or less acceptable for Rehima not to get marriage at her age?

R1. No, community not gives attention to girls' education. So people rather advise as every girl get marriage.

R6. No even if she is not willing to get marriage, parent can force her to get marriage.

R7. what so ever she do not want to get marriage, once somebody come and asked he for marriage, the accepted one is his idea only. Her denying have not acceptance. Because community perceive as if she loves him, may ashamed of to show her agreement. So may be due to shy that she denies to accept the marriage question. (laugh). Means very difficult to say I will marry him. That is why even if she loves him she says I don't want (laugh). So if once male wants to get marry no need of wait for her willing.

R3. If she is a student and not wants to marry, community could accept her. But if she is not a student and not wants to accept, her decision to get marry, will not community accept her; even if she is kid they will force her to get marriage. Community thinks if she is learning possible but if she is not learning only as she is wasting food so it is better if she gets married.

R5. The Community may take measure is that if she is not accepting get marriage, they can force her up o kick with stick to a make her get marriage. What if she is under fifteen-year-old, they will force her to get marriage. The effect on adolescent education especially for girls.

R3. If there is drought due to shortage money there is not fulfilled exercise book and pens, dress even food. Due to this is they will discontinue education even in both sexes. Especially male may go other town to get other income by working labor.

R3. If is there is drought no food at home; when she educate and returned to home she will not get food at home. Due to this like problem, she prefer to be absent from school.

R8. If there is shortage of food nobody will go to school rather they will go to search food from other area.

Especially male will go to search food from other town and female also may go to town to try business generating by trading so nobody thinks about education marriage at all.
