# Supplementary material for: Adolescent girls trapped in early marriage social norm in rural Ethiopia: A vignette-based qualitative exploration
Source: PLoS One. 2022 Feb 17;17(2):e0263987. doi: 10.1371/journal.pone.0263987 (PMC8853576; doi:10.1371/journal.pone.0263987)
Supplement: S1 File — (ZIP) [file pone.0263987.s002.zip › PDF FGD data/FGD 08_UAG_D2.pdf]

Unmarried adolescent Girl_District 2

**Discussants background information**		
Name Initial	Age	Education (Grade
MI	15	8
CH	12	7
DE	12	6
MA	12	5
HA	12	6
ME	12	5
UR	13	6
SE	14	7

Marriage Vignette

Ok, now I will tell you one story regarding marriage and you will give me your opinion and discuss on it then we will sum it up.

Rehima is a 16 year old student who lives with her parents. She attends school and helps her mother with household chores. One day Hindiya, Rehima's cousin came over to visit Rehima's family. They are are about the same age. Hindiya announces that she is engaged and getting married in a month's time. She also strongly suggests to Rehima that she should also marry soon as she is getting old for marriage. She reveals she also knows that there is someone from their village who is interested in marrying Rehima.

F: What would most Adolescent girls in Rehima's position do in this situation?

You know that Rehima is student, what you suggest girls like Rehima would do?

P7. I would say that Marriage is good for nothing just learn your education.

F: What is the real situation in your village? Do girls get married or follow your suggestion?

P7. They don't get married if they see one before marrying.

P4. Friends may advise them that such people are not good for her.

F: Tell me what most girls would do?

P7. If she has an interest on continuing her study, she will not accept the advice.

F: What would Rehima's friends and other girls expect that Rehima would do?

P1. They expect that she would marry.

F: Come on everybody, I need you to participate.

P8. They expect that she wouldn't learn then marry. It depends on her stand if she wants to continue her studies and be clever on her lessons she will not give attention to such idea.

F: What would girls like Hindiya expect that Rehima could do?

P7. If she says that she will not marry, she can continue her studies.

P6. It depends on her interest, if she want she can continue her studies or she can marry.

F: If she want to continue her studies, she don't listen anything about marriage, right? Let us say that Rehima decided not to marry at her early stage but what would Hindiya and other girls would say on her decision?

P1. If Rehima decided to learn, the girls would respect her decision and tell to the man who requested to marry Rehima.

F: But what is the interest of girls like Hindiya?

P1. Before they knew her decision they want her to marry but after her decision to continue her study, they will respect it.

F: If Rehima rejected the request for marriage, what would girls like Hindiya say to her?

P1. It's obvious that it is all about interest that Hindiya got married because she wanted to marry and she also should respect Rehima's decision to continue her study.

P7. Sometimes without any influence when her friend got married, she may also influence by her friends decision and get married too.

P1. Those girls who already started learning and experienced the advantage of learning would choose to continue learning but those who don't start learning would choose to marry. She shouldn't be influenced by her friends' decision.

F: In this village what is the maximum grade of girl's achievement?

P1. The maximum grade is 8th grade. Even those who completed 8th grade are not much. They are very few.

F: What about you? What would be your decision after grade 8?

P1. Continue learning and achieve the highest level or being doctor, engineer, pilot and etc.

F: Do your parents allow you to continue after grade 8th?

P1. No they don't.

F: Ok! Now Rehima decided not to marry, what would they do to her? What the challenge she could face? Can they convince her back?

P1. No they can't.

P8. No one can convince her.

F: Can they try to force her to marry by saying because they are married she has to marry. I mean is there peer pressure on this case?

P8: No there isn't.

F: Is there any support or acceptance for Rehima on her decision not to marry at this age? According to this village? What is the least and the maximum age of girls to merry?

P8. 12 – 18

F: What if she passed 18 years old?

P8. She has to marry before that.

F: According to your village what is the proper age of girls to get married?

P7. 12, even she can marry at her early 11th. If she get married at these ages she would be respected, but if she pass these ages without marrying they would consider her as a loser or has nobody to marry her. They don't respect her.

F: If a girl stayed until her 18th without marrying, what would the community call her?

P7. Good for nothing. But a girl who married at her early 11th would be in a problem more than the one who stayed until her 18th.

F: What kind of problem?

P7. Most of the time the problem occurs during her pregnancy time and maternity time, that's why it has said in the law women, should marry above 18 years old.
