# Supplementary material for: Adolescent girls trapped in early marriage social norm in rural Ethiopia: A vignette-based qualitative exploration
Source: PLoS One. 2022 Feb 17;17(2):e0263987. doi: 10.1371/journal.pone.0263987 (PMC8853576; doi:10.1371/journal.pone.0263987)
Supplement: S1 File — (ZIP) [file pone.0263987.s002.zip › PDF FGD data/FGD 09_MOAG_D2.pdf]

Mothers of adolescent girl's

Discussants background information			
Names initias		Education (Grade)	Age
1	AS	3	35
2	SA	4	35
3	WM	0	30
4	WA	0	30
5	NA	0	30
6	KY	3	35
7	KA	0	37
8	KM	3	42

Marriage Vignette

F: Now I am going to read you other history about a woman called Merima. Next I will ask you a question, so pay your attention.

What would most other mothers like Merima do in this situation? Ok, what do you say

P1 They can say Merima shoud refuse her daughter marriage

P2 Other mothers recommend Merima not to marry her daughter early

F Ok you, what do you say on this question?

P3 Merima should learn a lesson from the eldest that had moved back with her and not marry asha until she is young enough for marriage.

P5 Other mothers say to Merima, once asha clearly announce that she is not interested to marry' you also support her idea.

P4 What other mothers including those on coffee ceremony can say in this situation?

P7 Other mothers say her, why not you marry her? It is the right time to her to marry.

F What others of you say on this? What would other mothers in that area do in this situation?

P8 The eldest girl had moved back with you, this one also 15 years only. If she marries now she faces a challenge while delivery, can't manage her life properly and she also moved back with you.

P1 They say her, rather two of them move back to you do not marry the youngest one (Asha)

F Ok what you say on this issue?

P2 Her neighbors say her, ‘the eldest one came back to you and Asha do not want to marry so do not marry her until she is young enough for marriage’’.

P5 They can say her, ‘the eldest one divorcing her husband, so as a lesson you do not marry this one currently’.

F What woud most other mothers advise Merima to do regarding her daughter’s refusal of the proposal? What other mothers advise Merima to do regarding the refusal of her daughter to the proposed idea?

P6 Some of them say her, marry your daughter and some of them say do not marry her. Merima should accept asha’s decision.

F Ok other of you, what do you say on this?

P7 Other women say Merima, ‘’please marry her chat and girls better morning *[Seemed to smile].*

P4 Other mother Girls’ market is just morning, so you should marry her *[Seemed to smile].*

.

F If you say girl’s market is morning, what do you mean by? Explain it *[Seemed to smile].*

P3 Meaning, it is better to sell chat morning while collecting. Just marry if somebody comes to marry her, girl’s also so beauty at adolescent age so she should marry at that time *[Seemed to smile].*

P4 They mean that, it (marriage) is the right time for her to marry.

F Very good, you gave us a good idea. What others of you say on this?

P5 They say Merima; ‘do not miss her chance; you have to marry her now’.

P3 Asha say I should accept or fulfill my mother’s idea, her mother also aggressively decides to marry her. At this time she cannot manage the household, while delivery she may faces for many problems. If she will face a problem she moved back to Merima.

F Good, now I give a chance for those not get a chance, what do you think other mothers' advice Merima to do on this situation?

P5 Whatever people say it, until god do not say it

P2 If girls reach 15 years it is the right time her to marry. We would say to her "please marry you daughter... marry her, because chat and girls are best during morning"

P4 Everybody say her this is her chance, so marry her now

F Do you think that other people advice Merima to marry her daughter asha at age of 15? What do you think other kind of problems she may face? What do you say on this?

P5 Many different problems face her, if she gets pregnant, she encounters a problem while delivery.

P1 She can't manage the household property; she can't do anything by this age.

F Ok what do you say on this?

P3 They say that she is 15 years, chat and girls are at early. She also not matured for sexual intercourse. If she had a pregnancy, she encounters a problem while delivery and expose for fistula.

P1 Currently after government bodies thought us and health professionals also train us on early marriage. This day below 17 and 18 girls do not marry.

F Now I am going to read you a vignette additionally, so give me your attention. What most other mother say about Merima in this situation? Meaning, due to Merima supports her daughter's idea what other mothers say? What do you say on this?

P3 Those people who say her marry your daughter, after she changes her idea they regret

F What others of you say on this, what do you think other mothers say on Merima's decision?

P4 They appreciate Merima to accept her daughter's idea; they say her that is a good idea.

P5 If you were influenced by other mothers and marry her, especially by those with her on coffee ceremony you may face for different problems.If you were not support her idea; your daughter faces a problem. i.e you also face for a problem.

F What can others of you say on this? What you say on this, what other mothers say Merima that she support her daughter?

P1 They push her to change and marry her daughter

P1 They say; She should marry how you follow her decision

F Ok, what do you say on this?

P8 Those individuals who suggest Merima to marry her daughter asha will regret with their suggestion and say her later; ‘you do the right thing because she is not young enough to marry’.

F Is there additional or other idea? Ok what do you say on this?

P2 They advise Merima that her daughter is only 15 years, so she is not young enough to marry, you take the right decision because if you marry her by this age she may faces for many problems.

F What other people such as delala say about Merima?

P3 Delala say Merima; ‘do not miss her chance, just marry her.

P7 Delala/Broker tries to convince Merima to marry her daughter, because this is what he proposed before.

F Now I am going to the next question and it says, do you think that Merima’s decision is influenced by other people’s opinion and reaction?

P2 No one can change her decision, she generate that idea and reach on a consensus with her daughter, so she does not change her idea by any means.

P3 Since Merima and her daughter agreed up on same idea they do not change it.

F If no effect, what makes you think that way?

P4 If her daughter and Merima agree on same idea, the influential cannot change Merima’s decision.

F Once Merima and her daughter have a similar idea, plus Merima said ‘my daughter is only 15 age and Asha also sentenced that I am 15 year and do not want to marry now. So whatever the influence Merima do not change her idea.

F Would the opinions and reaction of other mothers make Merima change her decision about her daughter’s marriage?

P4 Yes, while she says that my daughter is not young enough for marriage, they influence her by different reasons.For example if somebody comes for some girl, the neighbors go there and decide with girl’s father to marry her even without mothers and daughter’s permition.

P6 As they mentioned there, they tell Merima a proverb which say 'girls and chat should be early'.

P2 In this community, there is a situation in which a neighbors and girl's father marry his daughter without considering her and her mother. I think they can convince Merima to change her decision.In addition, once they bring chat to girl's family, it is impossible to refuse. They say parent that this lady is not yours' only. Whether her family accepts or not, they decide that she has to marry, she is young enough to marry. *[Seemed to smile].*

F Do you have additional idea?

P3 Marring adolescents' girls by force is already past, while some body come and ask a girl for marriage, she is directly go to kebele administrative and report that her parent is going to marry me without my interest. After that the kebele officials arrest her family and take them to the prison immediately.

P4 You raise a good idea, but the question says that; can other mothers push Merima and change her decision?

P6 The influence of other people cannot change Merima's decision.

F Is there a condition or circumstances where it would be considered more or less acceptable for Asha not to get married at her age? Ok what would you say on this?

P7 The possible or acceptable condition is if and only if she is an age of 17 or 18 years.

P2 You know that she is 15 years of age currently, so is there a condition that acceptable if she does not marry at age of 15?

F At her 15 years of age?

P2 Yes, currently she refuses marriage, is that not? So, is there a condition that her refusal gets an acceptance?

P3 Yes, if her age is not young enough for marriage.

P6 Yes, if she is not willing to marry her decision get acceptance.

F Ok, what others of you say on this? i.e is there a condition that Asha's decision encouraged or get acceptance?

P6 Yes there is a condition that Asha's decision is encouraged and gets an acceptance. They say that 'she is only 15 years. Until she will be 17 or 18 age, she shouldn't marry'.
