# Supplementary material for: Adolescent girls trapped in early marriage social norm in rural Ethiopia: A vignette-based qualitative exploration
Source: PLoS One. 2022 Feb 17;17(2):e0263987. doi: 10.1371/journal.pone.0263987 (PMC8853576; doi:10.1371/journal.pone.0263987)
Supplement: S1 File — (ZIP) [file pone.0263987.s002.zip › PDF FGD data/FGD 10_FAG_D2.pdf]

**Fathers of adolescent girl/s_ District 2**

| Discussants background information | | |
|---|---|---|
| Name Initial | Education (Grade) | Age |
| 1) A.A | Uneducated | 50 |
| 2) H.A | Grade 3 | 35 |
| 3) M.H | Grade 5 | 40 |
| 4) A.M | Grade 4 | 36 |
| 5) A.A | Grade 3 | 45 |
| 6) B.A | Uneducated | 50 |
| 7) J.A | Uneducated | 45 |
| 8) J.A | Grade 4 | 40 |

**Marriage Vignette**

F: Merima is a mother of five. She has three daughters, of whom the eldest [age17] had moved back with her after divorcing her husband of 1 year. They all live in the same compound, including her sons and their wives. One day when all of them plus some mothers in neighborhood were sitting at a coffee ceremony in their compound, an intermediary (delala) comes to their house and tells them that Merima's younger daughter Asha [age 15] is being sought by a young man for marriage. Asha firmly announces that she is not interested to marry any time soon.

F: What would most other mothers like Merima do in this situation?

P2_ as many people are not educated here, most women in Halima's position make a deal with

Intermediaries to arrange the marriage

P3_ I do agree with the first speaker. We all collude and find a way to make marriage possible. This is an opportunity for her and we make sure she must not waste it. Marriage will continue unless she rejected the guy.

P4_ If she hesitates, everybody tries to convince her. No one rejects the marriage and suggests she should learn.

P5_They thought the failed marriage of eldest sibling who is back and currently living with the family is considered her own misfortune. This is Aisha's chance so they would say she should take it.

P6_When intermediaries come we collaborate with them and marry even girls who are 14 or 15 years old. Most girls in this area normally marry somewhere between the age of 14 and 18 years. If a girl could not find a husband in that age range, she will be called 'Haftuu' (meaning unlovable). Thus people say to girls once your time is over you cannot find a man so get married before that happens.

P7_If she insists to continue her education most people would say let her do what she wants. Others tell her not squander the opportunity to her disposal.

P8_ If she refuses the marriage because she dislikes the guy. People may agree with her for that day. But if she is firm no one can force her because doing so is illegal. Some section of the society advises her to use the opportunity of marriage. Your sister's fate is not your fate.

F: What would most other mothers advise Merima to do regarding her daughter's refusal to the proposal?

P1_In this rural society most mothers advice for the marriage to continue

P2_ Since our society in large are uneducated, they like her to accept the marriage. They tell the mother to advise her daughter to accept the marriage rather than in way she continued her education.

P3_ During the marriage day, the grandees try to pressure the family as long as they personal needs are fulfilled. Despite all the pressure, if Aisha continues to refuse nothing will happen to her for that.

P4_ Most people advice the family to refrain from taking place marriage through force because it is illegal. Other people advice the family to convince her than using coercion.

P5_ Most mothers advice her as her daughters not to miss the chance. But they cannot force them

P6_ . If the family dislikes the person who is seeking their daughter for marriage, they turn down the offer by using an excuse that their daughter is interested to continue her

education than marriage. They say that if you miss this chance you never get again so you have to marry.

P8_ Other people say advice your daughter not miss the opportunity, she refused it because she is young. To the contrary, other people advice the family to reject the marriage offer since Aish is not interested in it. These group, in addition brought into attention the earlier failed marriage of the eldest sibling. If they continue with the marriage they will be accountable in front of the law.

F: What would most other mothers say about Merima in this situation?

P2_since most people are illiterate, they would say she should get married. But if mother comes to terms with her daughter's position by refusing the marriage. The mother is seen as a role model.

P3_ he mothers probably say marry your daughter. If the mother does not like the family of the guy, they advance the idea that their daughter is interested in education. Once the issue of education is raised in the middle of negotiations for engagement the process likely stopped there. But if the mother has favorable attitude she want the marriage to go ahead even though her daughter is 14 years old. Now a day people slowly started to feel accountability before law. Earlier our decision was unanimous and final.

P4_According to this village marriage take place based on age limit. Usually girls between 15-18 years of age can be married. However, people more focus on appearance than actual ages as an evidence for maturity.

P5_the neighbor Applaud the mother for disallowing the marriage to take place.

P6_Grandmother sometimes can tell the family to stop the marriage by saying the girl is not matured enough. When the teacher reports to the local administration that a female student is forced in to marriage, the family will be fined. We go ahead with the marriage had it not been for the fear of the law.

P7_ First the mothers of the boys and girls make an agreement then the mother of the girl tells her daughter about the matter. If the girl here in this case, Aisha refused the intermediaries send other intermediaries to the girl' mother from the neighborhood by saying we like the girl but her mother has refused. If Marima still refused the proposal and my daughter is interested to continue learning. We do have such examples in our village. If both the mother and daughter are not interested in the marriage most people tend to support them.

P8_When the mother and her daughter in agreement refused, other from the local communities say leave them it is their business, they lost a good family, that was a good chance why she did not advise her daughter properly in time, what is the use to educate girls, and finally she will regret her decision by wasting marriage opportunity the mother can hear such discourse. If they insist other say be strong in your education.

F: what would other? People such as the delala say about Merima?

P6_ Intermediates insists her to get married.

P7_Intermediates establish a clandestine network that is tasked to change both Marima's and her daughter's mind.

P3_They tell the mother that the boy's family is good people and by refusing your daughter waste a good chance.

F: Would the opinions and reactions of the other mothers make Merima change her mind about her daughter's marriage? Would their reactions have an effect on her?

P8_Most would say we made a lot effort to change her mind but she refused, thus let her alone. Intermediates are not all the same but some have real convincing power and promise Mariama that Aisha Will continue her education despite the marriage in order to influence.

P7_In this there are some families who do not want to accept marriage offers while their daughters are attending education. They say I like to educate my daughter. Some ask the family how they refused the marriage offer, they responded that we want educate our daughter. Nothing happens to them. This is good we wish you as you like.

P6_ Once the mother and grandmother agree on the matter no one can able to change their mind. In this local area we believe that marriage cannot be successful without the blessings of the mother even if it took place. If the mother is not willing the opposing side less likely pushes the offer a head even the father would not overstep.

P2_No conspiracy if the family refuses. But they try to negotiate with the family through the use of influential people such as the daughters' uncles. Other than this, they do not have any option than stopping the offer.

P1_Mother and the daughter play a pivotal role. Neighbors may say marriage cannot hinder education

**F**: if no effect, what makes you think that way?

P6_People know the mother's role is decisive for the marriage to succeed even if the father is consented.

P1_Because the mother's and daughter's role is important in determining the marriage.

P6_Because it is illegal to force someone into marriage.

P5_Because there is a pressure from local administrators and teachers

F: Are there any circumstances where it would be considered more or less acceptable for Aisha not to get married at her age?

P3_Yes her decision is acceptable.

P5_her decision is acceptable if she either dislikes the person who seeks her relationship or wanted to continue her education.
P6_it is difficult to convince her if she categorically rejected the offer.

P8_ if she has no interest in her counterpart or else attending classes.

P2_it would be acceptable if she says I do not dislike the guy, but I like to continue my learning, he should also continue his education. Then we will be married having completed our education.
