# Supplementary material for: Adolescent girls trapped in early marriage social norm in rural Ethiopia: A vignette-based qualitative exploration
Source: PLoS One. 2022 Feb 17;17(2):e0263987. doi: 10.1371/journal.pone.0263987 (PMC8853576; doi:10.1371/journal.pone.0263987)
Supplement: S1 File — (ZIP) [file pone.0263987.s002.zip › PDF FGD data/FGD 11_FAG_D1.pdf]

Fathers of adolescent girl/s_ District 1

Discussants background information		
Respondent Name Initial	Age	Education (Grade)
P1. DB	48	2
P2. MS	32	5
P3. SE	58	7
P4. AY	40	6
P5. DA	37	3
P6. NA	49	7
P7. GK	36	8
P8. DB	46	4

Marriage Vignette

> Merima is a mother of five. She has three daughters, of whom the eldest [age17] had moved back with her after divorcing her husband of 1 year. They all live in the same compound, including her sons and their wives. One day when all of them plus some mothers in neighborhood were sitting at a coffee ceremony in their compound, an intermediary (delala) comes to their house and tells them that Merima's younger daughter Asha [age 15] is being sought by a young man for marriage. Asha firmly announces that she is not interested to marry any time soon.

F: What would most other mothers like Merima do in this situation?

P8. In such like situation the child have disputed, she knows about her daughter as she enough for marriage, the female age don't have less. Female do not have little. So that by that bad acceptance which they have practiced they influence that child rather than her what she say finding solution on what she say. There is no any who support that child.

P7. They cooperate together on she is saying am not yet mature but she is matured we give her for marriage and so they don't give any acceptance on what she say.

P6. She has no idea about her child, but the mediator came to that child with idea of mirage. Their highbrow says advise that child as our coulter she is doing as her mother going to face poor if that child disputes them on their idea. Culturally the highbrow has great roll. And also her mother has to take this mediator idea, also they think she should compromise on that idea.

P5. You should give her for them, as they take for her make ceremony of elder we help you.

P4. They make Aisha Mather in order her to say ok; they tell about all of that boy who going to marry her and they also give for her money and they say tell your daughter she will life. At this area coulter like that.

If it is her father they make him challenge by saying this one is our coulter after that they come to her. She should confirm such question. It's our coulter, isn't it?

P3. Culturally for example wither male or female before their parent recognize their maturity other say he/she matured for mirage. But they come to home your child have been matured so we give her. So they come to talk without you know nothing. They communicate on her then they ask you what matter is if you give her for that boy. If she says ok they will give her for marriage if not the come to them by elder form for the negation. So if she give to them permeation they will call him to her.

P2. In this case the mediator gets among them after that girl says ok. After she say ok, the mediator come to home and try to mediate also her mother to make her ok on that idea. If her mother may challenge them on that, they try to tell her more by say she is now 15 aged so she is matured. On that idea on which the mediator have been raised to her also the nigh brow also try to challenge as the idea is right to confirm and she has to marry.

P1. After a child aged 15 year she has to marry, if she loss the chance she find now, the next chance may not good for her so give her for mirage and compromise on it with her father. people would say, she has to go, she has to marry, it is time (she is already 15 years old) The mediator at first try to negotiate with her family by saying she may fair you so if you say ok, she also say ok. And mediator believe after her family confirm the idea she also say ok and if my family loved form it's no mater I will marry to him.

If a chance came for her she has to marry because the female is taken for marriage. They belief female is created for male so she has to join the chance she gat. So the mediator come to her mother to tells her more about the boy.

P8. The mother don't despot the chance come for her child, because she think if a chance loosed once, it's irreversible. And also she don't think the case of my child denied them is not seen to her as much. The people word is also not good after a chance available I give her. Wither the child also unaccepted her she try to challenge her by her fronds in order to they make her ok for the mirage.

After it's that tribe we give here at least they do not consider any problem that may come after the mirage. It's her responsibility, after her father and mother belief on that. While she doesn't go through the outside, her family can also give her by force. That means if they need that man.

F: What would most other mothers advise Merima to do regarding her daughter's refusal to the proposal?

P1. For to influence Asha to marry the man, they say as culturally your daughter is matured so give her for marriage, she should marry after she aged 15 year so give her. So they say to her mother don't lose this chance give her by advising.

P2. How she can denies your idea, influence her up to accept the idea. They motivate her mother in order to influence her for the idea.

P3. Their neighbors try to make her as she believe the idea for her to and make her as she get in promise. Then after she go for her child to make her as she accept the mirage question.

P8. They make her to marry, is she is out of your control, by saying you or the child is wiser than you so tell her and give fore mirage.

P6. After her mother and externally influenced she comes to change her idea and let for mirage.

Its mandatory they come to you with the chat, so you have to chow the chat with them that is a cutler. Wither the child confirms or not the elder come with required material. They came with same coffee, chat then wither they make the child say ok or not they don't back with the chat.

P3. At this time if the child is strong enough to deny their word they try to motivate her mother on her in order to influence her.

Let's return to the story. Merima listens to Asha and tells her neighbors that she respects her daughter's decision to delay marriage until she's older.

F: What would most other mothers say about Merima in this situation? What would other people such as the *delala* say about Merima?

P7. They don't influence her in other way but they motivate mediator to her child.

P4. They under line by saying she has to give her. This daughter is not only yours. She is not only yours but we have together. We are also responsible for her. If she continues to deny them they try to influence her up to taking for her bad attitude in surrounding.

P5. Since she is now accepted her daughter’s attitude so we have to make her out of our group and even punish her.it is not accepted as culture.

So she asks apologies as she made a problem to the surrounding and she punished by money and buck to her neighbor. Due to, she deny marrying her child.

P6. Her child said I am yet unmetered for mirage so she deny what they say, but the social influence their child by saying matured.

P5. When mediator go and consult them, mother and daughter cooperate together and said she is not matured for marriage.

So he try to tell them that bay is the son such like mane he is dignity man, they have such like luxury and so the friend of Asha mother friend also try to make her say it ok.

So this is for respect; if it’s the matter of education, he can make her to finish her class. He can also make for you such like goodness so you should give (marry) her to him.

P4. The problem is the same as which done over mister Mohamed, they can’t do anything except make her out of their member. That means there is person who work over mediation in that local, they judge over the child as they enough for marry without their family recognition that and come to their family and try to make them to understand matured child marriage. If that mediator says that one is good, they make what the man say.

And the community came to challenge the family as they have to give a child for that man. Then the community cooperate together by saying he has what to say by calling the elder at one place.

P8. The idea that Asha and her mother compromised the mediator try to challenge them by saying its good if you give her to him he is the son of dignities man, they have this and by say the like he try to challenge them. Otherwise he goes to tell the community manager and he also try to make her to say ok. Also if she doesn’t allow them by this form, they call to them all community members and try to challenge them as they should give the child to the man.

F: Would the opinions and reactions of the other mothers make Merima change her mind about her daughter’s marriage? Would their reactions have an effect on her? If so, what would be the effect? If no effect, what makes you think that way?

P1. Yes, at where she life if all person talk about that marriage it may, because the mediator man nominate same person at where she can be. All of them think what he need and also talk to her about that always.

P2. Yes, they can change her idea; all community has stand against her so she can’t do anything by her only.

P4. If she says I can’t change my idea, she can be displaced from the area with her child. But if the mother also displaces before them, they find a daughter from anywhere she able to go and take for the man.

F: Are there any circumstances where it would be considered more or less acceptable for Asha not to get married at her age?

P5. At this time, she says I can’t marry, she may too small, the man may be bigger than her if they are inappropriate by age.

P6. If she has knowledge, about badness of early marriage and its consequences of it at child.

P8. If wise man gets in the marriage this child may face different difficult, sexual inter cores, the problem during the child birth if the man support her what she say is correct.

P4. If part of law interrupts the mirage, by saying one female have to learn rather than marriage.

So the school has to support her.
