# Supplementary material for: Adolescent girls trapped in early marriage social norm in rural Ethiopia: A vignette-based qualitative exploration
Source: PLoS One. 2022 Feb 17;17(2):e0263987. doi: 10.1371/journal.pone.0263987 (PMC8853576; doi:10.1371/journal.pone.0263987)
Supplement: S1 File — (ZIP) [file pone.0263987.s002.zip › PDF FGD data/FGD 12_UAG_D1.pdf]

Unmarried adolescent Girl_District 1

Discussants background information		
Name Initial	Education (Grade)	Age
1. KM	8	14
2. KM	6	13
3. AS	6	12
4. ST	7	12
5. LM	7	12
6. MsH	7	13
7. HT	8	14
8. SM	8	16

Marriage Vignette

Rehima is a 16 year old student who lives with her parents. She attends school and helps her mother with household chores. One day Hindiya, Rehima's cousin came over to visit Rehima's family. They are about the same age. Hindiya announces that she is engaged and getting married in a month's time. She also strongly suggests to Rehima that she should also marry soon as she is getting old for marriage. She reveals she also knows that there is someone from their village who is interested in marrying Rehima.

What would most adolescent girls in Rehima's position do in this situation?

R1 Most adolescent girls in Rehimas position would accept the suggestion of marriage because of several factors.

R2 Girls like Rehima believe that refusing the suggestion of marriage and deciding to continue with their education is like wasting their fate and may subject them to lack husband in the future. Most adolescent girls would accept marriage proposal.

R3 … Refusing is like wasting their fate, they may not get the chance again. In addition, they may feel refusing would offend their friends.

R4 She may take the advise as a good thing and decide to get married

I What would Hindiya and most other girls expect Rehima to do in this situation?

R5 Most other girls expect Rehima to get married and cannot try to advise her to change her mind as it may look like a jealousy.

R6 Since the majority of her friends advise her to get married, it would be very hard for me to tell her not to get married, therefore I advice her to get married.

I But Rehima doesn't want to marry young. She announces that she does not want marry at this age.

I What would Hindiya and most other girls say about Rehima's decision?

R8 Hindiya may get offended or keep on trying to convince Rehima, while most other girls think that Rehima wasted her chance foolishly and made a wrong choice.

R7 They would repeatedly try to convince her, they may say 'he loved you why you ignore 'we've never seen girls benefiting from education'.

In a long run it is likely that they can make her change her mind.

I Would the opinions and reactions of her peers make Rehima change her mind about refusing the marriage?

R6: This depends on Rehimas strength because in this area it is common and believed that girls better get married than going to school. Therefore peer pressure is likely to be intense on Rehima and even if she kept on refusing there are consequences like isolation and defaming.

I Are there any circumstances where it would be considered more or less acceptable for Rehima not to get married at her age?

R2: Yes, if Rehima's parents are educated or at least want her to continue, if there are other relatives who are conscious about education and her teachers stood by her and motivated her it would be acceptable.
