# Supplementary material for: Adolescent girls trapped in early marriage social norm in rural Ethiopia: A vignette-based qualitative exploration
Source: PLoS One. 2022 Feb 17;17(2):e0263987. doi: 10.1371/journal.pone.0263987 (PMC8853576; doi:10.1371/journal.pone.0263987)
Supplement: S1 File — (ZIP) [file pone.0263987.s002.zip › PDF FGD data/FGD 13_MAG_D1.pdf]

Married adolescent Girl_District 1

Discussant background information			
SNo.	Name initials	Education (Grade)	Age
1.	RK	5	17
2.	IA	6	16
3.	KZ	7	16
4.	SH	4	18
5.	FI	4	17
6.	SA	3	18
7.	SI	7	18
8.	TB	4	18

F: Next our discussion is about marriage in this locality, similarly we continued our discussion with the vignette.

Rehima is a 16 year old student who lives with her parents. She attends school and helps her mother with household chores. One day Hindiya, Rehima's cousin comes over to visit Rehima's family. They are about the same age. Hindiya announces that she is engaged and getting married in a month's time. She also strongly suggests to Rehima that she should also marry soon as she is getting old for marriage. Hindiya reveals that she also knows someone from their village who is interested in marrying Rehima.

F: What would most adolescent girls in Rehima's position do in this situation?

P1: Other adolescent girls advices her saying, since I will get marry, you shall also marry into and they makes her like the boy whom Rahima has chosen for Rehima for marriage.

She thinks saying, since my friend had got married, I will also get marry. Since my friend had been betrayed, she also gets marry saying learning and family's work will not benefit me. This is what she says.

If she is willing to learn and her family are capable to support her education, Rehima shall not get marry due to Hindiya had betrayed/got married.

For the reason that Hindiya Rehima's cousin advice, she can hear to her if she says her you can learn, I can make for you whatever you want, and get marry.

F: What would Hindiya and most other girls expect Rehima to do in this situation?

P2: If she says, I will get marry like that of her friend. Since her friend is ready to marry her friends and other girls expects her to get marry.

She may get marry due to her friend influence and advices about marriage But if she says; let's learn, they can continue to learn by leaving marriage.

They expects her to get marry like her cousin.

But Rehima doesn't want to marry young. She announces that she does not want marry at this age.

F: What would Hindiya and most other girls say about Rehima's decision?

P3: While they go to fetch water together, they make her to change her idea and she decides to get marry even they were spite each other before.

Even she had aim of education, she thought to get marry obligatory for she may lose a friend later while all her friends got marry. And her friends consulted her and make her to change her idea.

If she hadn't to their consent inevitably she decides later while she became alone.

F: Are there any circumstances where it would be considered more or less acceptable for Rehima not to get married at her age?

P4: In reality it is very uncommon that the girl will say no and stay unmarried…But if she is willing to learn, or mostly spites those who came to marry her, she shall inform the teacher. Then maybe he convinces her family and stops her friend who advices her to get married. And

then oh…(she paused for a moment then continued talking) then just maybe her refusing not to marry may get acceptance.
