# Supplementary material for: Adolescent girls trapped in early marriage social norm in rural Ethiopia: A vignette-based qualitative exploration
Source: PLoS One. 2022 Feb 17;17(2):e0263987. doi: 10.1371/journal.pone.0263987 (PMC8853576; doi:10.1371/journal.pone.0263987)
Supplement: S1 File — (ZIP) [file pone.0263987.s002.zip › PDF FGD data/FGD 14_MOAG_D1.pdf]

Mothers of adolescent girl/s_ District 1

Discussants background information			
S.No	Name initial	Education (Grade)	Age
1.	NA	0	30
2.	KU	5	39
3.	AB	4	30
4.	HG	7	30
5.	NK	3	32
6.	ZG	5	49
7.	KB	0	39
8.	MA	0	30

Marriage Vignette

Merima is a mother of five. She has three daughters, of whom the eldest [age17] had moved back with her after divorcing her husband of 1 year. They all live in the same compound, including her sons and their wives. One day when all of them plus some mothers in neighborhood were sitting at a coffee ceremony in their compound, an intermediary (delala) comes to their house and tells them that Merima's younger daughter Asha [age 15] is being sought by a young man for marriage. Asha firmly announces that she is not interested to marry any time soon.

F: What would most other mothers like Merima do in this situation?

P1: Other mothers, the younger one already divorced, she is not in marriage any more, and the family member of the mother is increased; *"why not she allowed the little one for marriage"*, people may say/reflect such idea. Now they are actually 5 in number, if the little girl gets married, their family numbers will be minimized. If she understands this situation she is living in, she will allow the marriage. They are what people actually say.

Marriage is allowed for the age 17/18, so other mothers may say; *"this lady is 15 years of age, do not allow her to get married, she is below the age of marriage, let her to learn"*

But some people may say; *"you have to allow this marriage, she is 15 years of age, even the girls less than this age can get married."* this is the idea of uneducated people, *"obviously you children are many, if the fortune of your daughter are good, then your will also be good, this is good*

fortune/luck your daughter have got, allow her marriage, she may be helpful to you, allow her marriage", this is also the idea other mothers most commonly raise.

P2 this one got married but not she got divorced, similarly marriage doesn't benefit her any more, you better let the lady start education. Marriage all about ending with divorce, that is all about it. The elder one is divorced and she is at home with her mother, but people actually say; "in life there is good luck also bad luck. One person's bad luck may be good for the other person" (She replied loudly), so other mothers allow marriage, even if Ashan is not willing to get married, this doesn't make mother happy, definitely she allow the marriage.

(Slowly replied), mother may not allow the marriage, because the age of Asha is not old enough for marriage, she is bellow age of marriage. If she gets married she can't deliver at this age. She can't also manage family.

F: What would most other mothers advise Merima to do regarding her daughter's refusal to the proposal?

P3: Logically, people could have advice Merima (The mother in the story) not to allow marriage because, she (her daughter) is below the age of marriage, but the reality is, no one advice her in such way. Based on our rural condition, other mothers' advice saying; "allow her marriage, allow it!!!" they influence her to accept the marriage and persuade her daughter to agree. (She replied loudly with motivation), *"just allow the marriage, she has got good luck, do not affect her good luck, she has got good luck do not push it back"*, this is how mother advice her. (Smile and continue), once they meet on chat/khat [1]ceremony, they advice and discuss on similar event and influence the mother to allow the marriage.

P4: the boy do have good behavior, he has good conduct, allow the marriage and accept his request", saying this they influence her to say ok, (she laugh...all other discussant also laugh)

(Discussants are laughing and make different voice and speak....,'this is right, this is right....' one discussant loudly dominate others and say; *"he has good conduce, he respect people he is also*

---

[1] Chat/khat: it green stimulating plant which is locally available. People ingest it to make fun get stimulated.

respected, hence allow the marriage", this is what people say and how they influence Ashas mother (smile...). People taking about the boy asking marriage have no idea about him, but they talk about his good behavior (smile...other laugh...)

P4: in our rural area, if mother allow marriage and give their daughter, they get clothing, dressing for mother, pant/trousers for father, and also shoe, others. This all are given for parents. When people join on chat/khat ceremony in the house advice mother saying; "the daughter is not yours, she ours, the daughter belongs to the group/sect/community, it is not only fathers who born her, she is ours..." when people thought like this; they made an agreement on her, the clothing given for the parents is also another benefit, this is all about parents are grateful for (laughing....)

F: What about you, your idea pleasE, (facilitator gave chance for the shy discussant. But she simply laugh without giving any single idea, *"I have no idea, what they have said is enough"*, she said.

P5: when elders collected in the house they talk to the mother and convince her; *"do not put your daughter in the sight of people, if you refuse inform of these elders, she may not get success in her future life, the chat/khat we have break is not good to her, please allow this marriage."* Saying this they her to say yes. If mother refuse, or father refuse they both can't decide on their own daughter. The elders talk strongly and made them change their mind; they talk, talk...talk and finally end up with girl's marriage.

F: Would you please explain what is meant by breaking chat/khat?

P6: The time elders come the daughters parent house, they come up with chat/chat. It is all about cutting and chewing chat/khat. They tell the mother that refusal of marriage after they cut chat is bad to the daughter. What do the parents do then; they get fouled and agree with the marriage.

P2: Yes. yea, once the elder people bring chat/khat and come to the house of daughters parent, it is called magantaa[2]. People call it magantaa. This means; *"if the parents do not agree, it i.e. believed that, either the daughter will die or she will not get success in her future*

[2] Magantaa: is to mean curse if someone or people do not agree with what they are asked to do or offer.

life, she will face bad luck, she better get married and she can continue her education", this what elders say to parents of the daughter. Once she gets married no one worried about her education, she does not continue education.

F: Please make it clear, it this actually the culture of people that most people accept and exercise?

P7: They all replied saying; *"yes this is our culture"*

Of all discussant one voice dominate and replied loudly, *"it is a lot, many girls get married from grade 8, they stop education for marriage, it is uncountable, it is not one or two, many, once the elders tell her parents about her school continuation, no one worried about her education, even if parents strongly refuse, there is no way to escape from the elders idea"*

F: Please tell about the community culture on the age of girls' marriage, it is not common to marry 15 years old girl?

P4: (She slowly replied), no marriage at this age, there is a *(smile...)(the other discussant laugh, with the idea)* if girls' marred below the recommended age of marriage, that individual or parents will be accused of it, is it not crime? (She laugh...the other discussant also laugh), for this reason if 15 years old daughter get married, it will kept secret. The one who get married at 15 years are thought she is 18 and above, which is not actually true (laughing...)

Mothers like Marema, who like in rural village like ours do agree, girls at the age of 15/16 get married.

F: Let's return to the story. Merima listens to Asha and tells her neighbors that she respects her daughter's decision to delay marriage until she's older. What would most other mothers say about Merima in this situation?

P5: *(Assertively replied), "the daughter is not yours, we can decide on her, did you born her alone, she is our daughter as well, we can decide about her marriage"* saying this, when elder people come the parents home to ask their daughter for marriage, neighbor decide and agree about the marriage of daughter, even if mother refuse, she can't.

(Loudly replied), if the daughter refuse and reflect her feeling, people say, “she do not know about this, she can’t decide, you have to advice what to do”. Her mother get convinced and advice to change the idea of her daughter.

F: What else, what do they do or say?

Other mothers and people advice and convince Marima saying; “after marriage she can continue education, she has to marry”, they strongly advice her as if the mother commit a serious mistake.

F: What would *delala* say about Merima?

P6: Delala do not care about anything; once he collect his money, once he/she is paid, he/she can convince people and let the girls marry.

(She assertively replied), he persuade Ashas mother, he cajole her strongly. He also involves other person to convince her. For instance he went to Ashas neighbor and informs her about the issue. Her neighbor then talks to Ashas mother, she advice and persuade her in different method. Delala then arrange to bring both bride and groom together for discussion. Probably they may go together which is called jala deemaa. Finally when the issue is handled by elder people, Ashas father strongly refuse, but her mother is informed about it and she keep quiet, do not strongly refuse.

P7: since her mother is pre-informed about eh issue, she do not refuse, she keep quite. Then delala take the daughter and let her to go with the boy, the one who want to marry her, what even her father talk about the mother is aware of it and she do not say ay thing, that sis it (laughing...others also laugh...)

P3: when her husband gets annoyed and ask her about the issue she replied to him saying; “do you think I am aware of such issue? I do not know about it, today she left home to bring material from neighbor, I do not know where she go, the reality is she know eve thing (laughing...).

F: Please tell us where does the girl actually go?

P8: they both discuss on day time and they go together. She goes with the boy. She goes with the one who want to marry her. Once the boy takes her to his relative’s house, he informs his elative, probably his uncle. When the daughters’ father is back from work and he realize his

daughter is not at home he ask his wife; *"where is she"*, his wife also reply; *"I have no idea, I think she went out to Ms 'X' house to bring something".* Immediately, the issue will be told to the neighbors and they inform the daughters' parents about the issue, her father actually gets annoyed and keep till morning. Culturally in the morning the elder people sent to the daughters' parent, they bring chat/khat. The elders inform about the issue to the parents and they open discussion for negotiation, they complete all requirements and eat prepared food and complete marriage issue (laugh...)

The father of groom ask his neighbors for support, I inform them that his son took somebody daughter for marriage saying: *"my soon bring a lady for marriage, please bring this chat/khat to the bride parents for reconcilement"*. People with elder people once they reach the groom parents' house and put down the chat/khat, they start negotiation. The father of the groom strongly annoyed and refuse, but mother keep quiet, because she know it, then the elder people give 1000birr for the bride parent for reconcilement, the bride father take and keep the money with him. Next another 1000birr added as a support. Totally 2000birr is given for the bride parents that are all. The ceremony then comes, shuuroo, marqaa[3], oil for cooking, they eat and go apart. The bride and groom then start living together.

Till the elder people bring char/khat to the bride parents and the reconcilement process finalized the bride and the groom can't sleep in same house. They both stay apart and go for examination[4]... *(The other discussant opposes this idea and interrupts her...as if to say no such culture...no examination...there is no.....there is no....no....before there was, it is not now.....)*

F: (*Facilitator settles the condition and let discussant to reflect idea one by one without opposing any ones idea*), please tell us turn by turn.

P6: As to my knowledge, the bride and groom are not allowed to sleep in same house till they get examined and their blood condition is known. The elder people also make sure they get examined, the look at the paper sent from health facility.

[3] Shuuroo, marqaa: Are the locally available food types, for instance marqaa is to mean porridge.
[4] Examination: is to mean HIV test before marriage

The other feeling she experience is, since things are going out of her expectation, she worried a lot.

F: What else any other idea?

P7: It all this, examination is not common.

F: Ok, come to our point and let me remind you, Marema agree with Ashas idea and she refuse about her marriage? What do other mothers do say? Please tell us any additional idea.

P:2 Some mothers may say; *“let me ask the interest of my daughter, if it is her interest no matter but if it is not I do not allow this marriage”*

F: How do you think is the reaction of other mothers affect Marema, because they all advice and influence Marema to convince Asha for marriage?

P5: (Uninvited discussant replied loudly), yea, Marema may change her idea; she may regret and change her idea. Since majority of people push her to allow marriage, she change her idea and allow marriage.

Dallala is strong to change the idea of the mother, since Delala is strong enough to convince the girl and other people and are not passive to convince mother. She simply changes her idea and start convincing their daughter (laughing...), she then feel stressed from people pressure.

F: How to these affect marena?

P4: Is it possible to stay out of the neighbors’ interest, there is no way to refuse and debate the idea of neighbors. She can do what her neighbors need from her. She prefers Asha to get married and get divorce if the life goes wrong after marriage, than refusing the elders and neighbors request. She may think this way.

Nothing will happen to her, probably if her daughter strongly begs her mother to refuse marriage, she may refuse, but this is rare. She may be disturbed, he mind can’t be stable. Since she allows marriage by force she disturbed.

If the bride fails to lead peaceful life with the groom, she may divorce, if she is not ok with life she divorce, she go back to her mother house. Since she is divorced, elders can’t enforce her like it was before.

F: Are there any circumstances where it would be considered more or less acceptable for Asha not to get married at her age?

P1: If Asha present her case to teachers; “my parent enforce me to get married, without my interest”, this is the way she can present her case. The teacher then brings the case to legal norm and Asha may be saved from marriage. The teacher accuses her parents.

It is only teachers that can respect and accepts Ashas idea.

F: What else, please tell us you idea (shy discussant invited)

P3: The daughter’s parent, neighbors all agree on the marriage of Asha without her consent, if she leaves the house and went to town and start life she may not get married. If they can’t find her, and can’t find her they stop talking about marriage. In this way she may work with people as a servant and continue education at night. Some daughters took such measure.

F: Please tell us any possibility for the marriage refusal of Asha gets accepted without leaving her parents house.

P: There is no way, once she girls I proposed, whatever the condition is, unless she leave home or inform her teacher for legal issue, no way to accept the marriage refusal of Asha. No way.

Other discussantS said, “yes” in one voice
