# Supplementary material for: Adolescent girls trapped in early marriage social norm in rural Ethiopia: A vignette-based qualitative exploration
Source: PLoS One. 2022 Feb 17;17(2):e0263987. doi: 10.1371/journal.pone.0263987 (PMC8853576; doi:10.1371/journal.pone.0263987)
Supplement: S1 File — (ZIP) [file pone.0263987.s002.zip › PDF FGD data/FGD 15_UAB_D1.pdf]

Unmarried adolescent Boys_ District 1

**Discussants background information**		
Name initial	Education (Grade)	Age
1. MT	6	14
2. CY	8	14
3. MT	7	15
4. CM	8	14
5. GU	8	14
6. AB	7	14
7. WA	7	16
8. SA	6	15

Marriage vignette

I *I will tell you a story of a girl I named Rehima (that is not her actual name) living in this woreda. I would like you to listen to the story carefully and discuss the questions that follow*. Rehima is a 16 year old student who lives with her parents. She attends school and helps her mother with household chores. One day Hindiya, Rehima's cousin came over to visit Rehima's family. They are about the same age. Hindiya announces that she is engaged and getting married in a month's time. She also strongly suggests to Rehima that she should also marry soon as she is getting old for marriage. She reveals she also knows that there is someone from their village who is interested in marrying Rehima

I What would most adolescent girls in Rehima’s position do in this situation? Is the history clear

D1 Yes , all discussant shows their agreement by nodding their head

D2 One of the discussant replied that, as she got that loves her, she got married. In this community they accept hindiyas advice.

D3 she accept and marry as hindiyas advice.

I Uhhh ….. Any more please?

D4 while Hindiya informs rehima, rehima may discuss with her friends and may refuse Hindiyas advice and may continue her education.

D3 when hindiyaa tell her she can refuse. She has to continue her education and get married after she reaches in higher condition. So she did not receive her advice.

I Ok very good. Now can you tell us about the real things that are found in the community? If such condition happens to adolescent girls how can they respond?

D3 (Continued saying)…in our community adolescent girls like that of rehima did not accept such advice. Rather they continue their education.

I At what age did adolescent girl married in this community?

D2 in these community adolescent girls married around the age of 16.

I So rehimas age is 16 why she did get marry at this age?

D2 Continued saying……Uhhh….even though her age is 16, if she is a student she can marry later.

D7 Other discussant said as some girls may not accept hindias advice, but most of the girls accept hindiyas advice and goes married. Continued saying she do not accept hindiyas advice rather she has to continue her education.

I Uhhh …please other

D4 she can accept hindiyas advice and marry him. Because if she refuse her advice she fear that people may say her *"Carraa kiyyaa nii darbaa, haaftuun nan jadhamaa jattee ni fudhatii. Literally it means that may lose my chance, and may be called as not needed …so she can marry him.*

D2 once she discussed with her friend hindiya she will get married soon. As hindiya also discuss with her as she had also a friend to marry soon, rehima will decide to marry soon.

I Uhh.... Can you add one more?

D3 After rehima discussed with hindiya she will decide to marry him because of fear of called left over. Hindiya can advice her saying am on the way to marry soon….during this she think over it. Then immediately she will decide as her age was gone and as she has to marry soon.

D5 after rehima discussed with rehima, she ignore her advice and continue her education. Finally, she will decide to continue her education to grade 12 and college. Later she will marry.

D4 some individual accepts her advice and some may not.

I What would Hindiya and most other girls expect Rehima to do in this situation?

D7 a girl has to accept (the marriage proposal) and get married, unless she wants to be called 'Haftuu' and risk to be unmarriageable".

I At what age do adolescent girl marry in this community?

D3 in this community mostly adolescent girls married at the ages of 13-15 years. But there is also who marry at the ages of 12.

D8 Continued saying, even though rehima is 16 years she can marry after she complete her education.

I But Rehima doesn't want to marry young. She announces that she does not want marry at this age.

What would Hindiya and most other girls say about Rehima's decision?

D3 they cannot say anything. But if she quarrels with friends they said, *haftuu*. Because she rejects her advice.

D4 One of other discussant replied as ...They cannot say anything. Once she refuses their idea it is her right.

I in this community what did they say on her decision?

D5 they insult her saying "haftuu". Continue saying..as the culture of this community your age is going on…after while continue saying *Attii kannaa boodee nii duuloomtaa. Literally it means you are going to be old.* They also insult her saying you will remail at home.

D6 ok she is already refused her advice, and they consider her as she is foolish. Continued saying…occasionally they may quarrel each other. During this they insult her saying haftuu.

I Uuhh… any other

D7 One of the discussant replied saying, since she refuses her advice they can say you do not want to marry early. You will marry when you get old. During any disagreement they insult her saying *haftuu*. Continue saying they insult also as you will marry an old man.

I What do you mean when you say early marriage?

D8 Continue saying…Early marriage is a marriage who marries at the age of less than 16 years.

I Would the opinions and reactions of her peers make Rehima change her mind about refusing the marriage?

D1 One discussant continues replying as, regarding to hear feeling about their reaction, rehima can change her decision and got married. Because of their insult she can change her decision. And got married.

D2 Another discussant continued replying as some of the adolescent like that of rehima may no change their decision if they are active enough in their education. Rather they continue education.

D3 …Sometimes if fighting or disagreement is created in between, they may insult saying haftuu. This can affect her feeling and forced to think over it. Later she can marry without continuing her education.

I Please can you add more on this

D4 all of my friends were married, all of them had child, if we quarrel the insult me *saying haftuu, dulloomtuu*… so she decided to get married.

D5 One of the discussant continued saying, if she refused to accept what they said…her friend says she need only her education other than else. Finally they ignore her.

I Uuh …you

D6 As my friend says her friend replied as she did not receive what other person says to her, she is selfish; she did not accept our advice so we ignore her.

I Thanks very much. You have told me so many interested things. Now continuing this are there any circumstances where it would be considered more or less acceptable for Rehima not to get married at her age?

D7 the chance that she had, even her friends advice her to get married early, she knows more than those others, so she can decide by herself and got married later…she can marry after she get in a better position means after she complete her education.

D1 Another discussant replied loudly she cannot get married at this age if she is a student and if she wants to continue her education.

D8 she can married after she complete her education because she had better outlook than her friends.

I Uhhh …please other

D3 if her parents want her to continue her education, she can be safe from marriage. Continued saying...if she changes her place from where she is learning now and continued her education in other place she can lead better life than those married early by dropping out their education.

D4 if she decide to continue her education and underline as if she get marry when she reach in good position nobody can interfere with her idea.
