# Supplementary material for: Adolescent girls trapped in early marriage social norm in rural Ethiopia: A vignette-based qualitative exploration
Source: PLoS One. 2022 Feb 17;17(2):e0263987. doi: 10.1371/journal.pone.0263987 (PMC8853576; doi:10.1371/journal.pone.0263987)
Supplement: S1 File — (ZIP) [file pone.0263987.s002.zip › PDF FGD data/FGD 16_MAG_D4.pdf]

Married adolescent Girl_District 4

Discussants background information		
Name Initial	*Age*	*Education (Grade)*
RA	*16*	*0*
AM	*17*	*0*
IM	*16*	*Grade 5*
IA	*16*	*0*
IU	*16*	*0*
DU	*15*	*0*
KM	*16*	*0*

Marriage Vignette

F: Rehima is a 16 year old grade 8 student who lives with her parents. She attends school and helps her mother with household chores. One day Hindiya, Rehima's cousin came over to visit Rehima's family. They are about the same age. Hindiya announces that she is engaged and getting married in a month's time. She also strongly suggests to Rehima that she should also marry soon as she is getting old for marriage. She reveals she also knows that there is someone from their village who is interested in marrying Rehima.

What would most adolescent girls in Rehima's position do in this situation?

F: Did you understand the story?

There is this girl called Rahima. She is 16 year old grade 8 student and now she is living with her family helping them by work so that she can continue her studies. One day her friend Hindiya came by to visit her and she told her that she is already engaged and planning to get married next month then she also advised Rahima to marry because her age is passing by and even there is a guy who wants to marry her.

R1. May be if she meets the one who wants to marry her, they can make a deal and

discuss with the neighbors.

F: I need you to put yourself in place of Rahima. What would you do if you face such strong peer pressure?

R2. I will tell him that I would learn.

F: What would most girls do if they face such thing?

R3. She may talk to the man and if they agree they may send a broker to her village.

F: What do you mean by a broker? Look, you are telling us general idea as if we live here with you, but we came from different culture and area and we need detail information because we need to know.

R3. It is just when they came to her place to negotiate with her family and elders of her village.

F: What do you mean?

R3. It is just they have to ask for her parents' and elders of her village's permission.

F: Look, no man approached her but her friend just told her that there is this man who wants to marry her or like her. That's all. It is just a peer pressure.

R3. I did understand, but according to our area's tradition, if a man has any intention towards to one girl then he must approach her and ask her for marriage. And if she is willing to marry especially if her age is up she will decide to marry him as well.

F: What is the right age to get married according to your tradition?

R6. In between 15 and 16 is the right time according to our village.

R7. As of me, I will look into both ideas which are education and learning. After I listen to my friend's idea or advice then I will go home and sleep over it then decide what to do.

F: This is your personal idea, right?

R7. Yes

F: But I need you to tell me the tradition of many girls in your village. What would be their decision?

R7. It would be marriage.

F: What is the maximum age for women in your village to be married?

R7. The maximum is 17

F: Aren't girls who pass their 18?

R7. There are, of course.

F; What would they call those girls?

R7. Just girls

F: No, that's not what I am asking, isn't it difficult for her in that village?

R7. They call her big girl.

F: Doesn't this hurt her in any way?

R7. No, it doesn't.

R6. You know according to this village, marriage has more value than education. So, many girls will easily influenced by such kind of peer pressure.

F:What would other girls like Hindiya expect from her to do?

R6. Few people may advise her to continue her education and not to accept the marriage idea. But there are a lot of people who will force her to got married and leave her education.

According to this village what is the community's and family's interest?

R6. Their interest is seeing her in marriage.

F: As I can see Most of you are illiterate, why is that?

R6. It is because we quitted learning.

F: Is it because you got married?

R6. No, it is because I had no one to send me to the school because I lost my mother and father.

F: Let say that Rahima finally decided to continue to her education and told to her friends and Hindiya that she doesn’t want to marry, what would be their response?

R6. If she does not accept what we (peers) advise her, (to accept marriage), later she will remain at home and become “haftuu”. This means that she is leftover… unmarried and stays at home forever.

R7. They may force her to get married but she will stand firm on her decision.

F: Is there anything that they can do on her because she refused to accept her idea?

R7. I don’t think so, maximum they may try to convince her but if she keeps refusing them, they will let her to learn.

R4. If she refuse their idea and keep learning they may discourage her that she is struggling for nothing.

F: Any other thing they can do on her?

R4. Nothing else

F: Now, we need you to tell us how the marriage process goes in your village? How was it back then when you got married? How do people date each other in your village?

R6. Most of the time, there are brokers or intermediate that bring the man and woman to each other.

F: What intermediate?

R6. It is a person, who introduces the man and the girl to each other.

F; What would the man do when he wants to marry a girl?

R6. Most of the time he sends one person to her to convince her for him and that person will convince her and brought them to each other.

F: What about elders?

R6. Yes! They will also send elders and the elders ask the family and if the family's response becomes positive they may marry each other.

F: Do they send elders before date or after?

R6. They can send for both.

F: Ok! What else they would do? Any other way that the man and the woman start dating?

R5. Sometimes they start living with each other and after sometimes they send elders to both parents. Actually, there are people who send elders before their date.

F: Which one is more practiced in your village?

R5. They are those who send after they started living with each other.

F: Any other idea? What kind of negative effects can be on a girl like Rahima just because she decided to learn instead of marriage?

R5. It may hurt her psychologically.

F: How?

R5. People's ignorance and criticisms will affect her badly.

F: Is there any way that these ignorance and criticisms may change her stand?

R5. No, if her decision is a true decision she won't quit her school.

F: I need you to tell me the majority who will survive these ignorance and criticism in your village?

R5. It depends on that person. You can't put a general aspect.

F: So, you are saying that there are girls who would change their idea because of these ignorance and criticism?

R5. Yes, of course.

F: What about if her parents force her to marry, would she marry?

R5. Yes

FYou told me that a girl marries in between her 15th and 16th years of age, right? Then what will happen if she passes these ages without marrying? This is the last issue that we are going to discuss on it.

R3. Would you repeat what you said?

F: If a girl wants to marry after her 17th, 18th or 20th, will there any means for her?

R3. If she becomes 16 while she is learning, she will continue learning.

F:If she is learning, right?

R3. Yes

F: Fow can one woman stay without marrying a man?

R4. If she stay without marrying and completed her school then she can marry an educated person like her.

F: Is it acceptable by the community?

R4. Yes

F: What else?

R4. If she accepts the idea of marrying she will and if she refuses no one will force her.

F: Does the community have a belief that a girl shall not marry because she is learning?

R4. Yes

F: Out of learning, is there any other reason for woman not to marry?

R2. If she doesn’t want to marry I mean if it is not her interest she can live without marrying.
