# Supplementary material for: Adolescent girls trapped in early marriage social norm in rural Ethiopia: A vignette-based qualitative exploration
Source: PLoS One. 2022 Feb 17;17(2):e0263987. doi: 10.1371/journal.pone.0263987 (PMC8853576; doi:10.1371/journal.pone.0263987)
Supplement: S1 File — (ZIP) [file pone.0263987.s002.zip › PDF FGD data/FGD 17_UAG_D4.pdf]

Unmarried adolescent Girl_District 4

Discussants background information		
Name initial	Education (Grade)	Age
1. SA	5	14
2. CHN	8	16
3. SK	6	15
4. DD	4	14
5. DB	6	14
6. CHA	3	13
7. KK	5	13
8. MA	4	13

Marriage Vignette

Rehima is a 16 year old student who lives with her parents. She attends school and helps her mother with household chores. One day Hindiya, Rehima's cousin came over to visit Rehima's family. They are about the same age. Hindiya announces that she is engaged and getting married in a month's time. She also strongly suggests to Rehima that she should also marry soon as she is getting old for marriage. She reveals she also knows that there is someone from their village who is interested in marrying Rehima.

F: What would most adolescent girls in Rehima's position do in this situation?

P1: The time of education may pass over but the time of marriage does not. As to my idea based on the information you have told us, in this locality other girls like Rehima decide to get marry. Because her relative of same age is on the way to marry; hence when her friend advice her saying, you are close to getting old for marriage, this made her to think about marriage. Daughters' advice each other in this locality; one the girl goes for marriage, there is no way to continue education. This is common in this locality.

If the girls is not on education, the advice from friends are strong, they say; *"once you have got the chance you have to marry, if you refuse to marry, no one will marry you after you get older than age of marriage, this is right time, your friends are getting married"* (laughing...). Then she easily convinced to get married. Even if she is on education, other daughters consult each other saying; this education doesn't bring change in life, no change can be seen you have to marry. Very rarely, some daughters refuse and continue education; this is the fact of our locality.

P2: Now, she (Hindiya) come and told to her about marriage, she inform her that she is going to marry, she is discouraging school continuation, this make and motivate Rehima for marriage. Once Rehima realized that her friend is ready for marriage, she also gets motivation and follows her friends' consultation. Even Rehima is afraid of the age of marriage, she fears to get older than the age of marriage.

P3: In our locality if one girl gets married and the other one is advice for the existence interested boy in the locality to marry that girl, she immediately decide and g for marriage. She then stops education.

F: What would Hindiya and most other girls expect Rehima to do in this situation?

P4: Stopping education and getting married; this is what other daughters expect Rehima do.

P5: Yes, as to my idea, other daughters expect Rehima to accept the advice of Hindiya and ready for marriage, no one expect her to refuse marriage and continue school or education.

Do you have different idea (facilitator ask the discussant and they all say "no"),

P6: once the fortune of marrying comes in life, girls should not refuse but immediately go for marriage

F: But Rehima doesn't want to marry young. She announces that she does not want marry at this age.

F: What would Hindiya and most other girls say about Rehima's decision?

P7: Rehimas friend actually insist her to decide and get marry, but if she refuse and do not want to marry, they do nothing. She continues education.

P8: (smile and continued), Hindiya is advising the girl to marry, this is from self experience, if Rehima strongly refuse marriage, her friend (Hindiya), get offended on Rehimas decision, she do not talk to her, she stop having communication friendship with her. Rehima feel happy only when she is successful with education. For example if he continue education by ignoring he reaction of her friend, finally the life of the two girls will be different, Hindiya will have children and start leading rural life while, Rehima can be employed and live in urban area. In this case Hindiya regrets for the advice and reaction she has shown to her friend. The reaction of other daughter on Rehimas refusal may be they get offended, reaction may be difficult.

P2: I personally stoop having friendship with her. Her also friends get irritated; they do not continue friendship with her. In this situation if national examination fails, she (Rehima) will regret for not accepting the advice of her friends, she feels shame, she lose hope for marriage, because all her friends are already married.

F: Would the opinions and reactions of her peers make Rehima change her mind about refusing the marriage?

P5: Yes, definitely, the repeated advice frequent advice from friends can make Rehima to change her mind.

P6: I have also same idea, the friendship between the two girls may stopped for the time being; this make Rehima to Change her mind and agree for marriage.

P4: Actually when friends stopped friendship with Rehima due to refusal, she agrees and maintains her friendship with other girls.

There is no reason for Rehima to strongly refuse marriage, because these girls stay together most of their time, they meet each other every day, and hence if Rehima do not change her idea on today, she can be changed on the next day.

P3: The marriages of girls are decided by girls, with the influence of friends and boys. Parents do not enforce girls to marry, due to his the ideas of girls can be changed from the influence of peers. What even the marriage condition is, unless she love the boy and willing to marry, no one can in force her.

F: Are there any circumstances where it would be considered more or less acceptable for Rehima not to get married at her age?

P2: If the girls inform her parents and probably her parents help her she may not marry.

F: Any more ideas?

P2: No, way, she decides herself, if she changes her mind from the influence of parents and friends she may not marry. Sometimes parents with their daughter to marry on time.
