# Supplementary material for: Adolescent girls trapped in early marriage social norm in rural Ethiopia: A vignette-based qualitative exploration
Source: PLoS One. 2022 Feb 17;17(2):e0263987. doi: 10.1371/journal.pone.0263987 (PMC8853576; doi:10.1371/journal.pone.0263987)
Supplement: S1 File — (ZIP) [file pone.0263987.s002.zip › PDF FGD data/FGD 18_FAG_D4.pdf]

Fathers of adolescent girl/s_ District 4

**Discussants background information**			
Name initial		Education Level	Age
1	AA	4	40
2	SH G	9	38
3	SM	0	42
4	MY	5	31
5	AM	0	35
6	GA	4	49
7	NA	6	40
8	AA	0	70

Marriage_ Vignette

F: This is a discussion held between elders. Now we will start this discussion. Merima is a mother of five. She has three daughters, of whom the eldest [age17] had moved back with her after divorcing her husband of 1 year. They all live in the same compound, including her sons and their wives. One day when all of them plus some mothers in neighborhood were sitting at a coffee ceremony in their compound, an intermediary (delala) comes to their house and tells them that Merima's younger daughter Asha [age 15] is being sought by a young man for marriage. Asha firmly announces that she is not interested to marry any time soon.

What do you think other Mothers like Marima can do in this situation?

P1: They can do nothing about the one who is already married, and will discuss about those who are left.

F: Let's us not get into discussion on the one who is already married, but focus on what they should do about the little girl. What do you think other mothers like Marima can do about Asha's refusal to get married?

P2: There is an Oromo saying which says 'a girl's marriage before the age of 18 is not considered as marriage'. (Seemed happy)

P3: She doesn’t know how to take of the house. It’s also true for a boy, who gets married before the age of 20. so that other women support their daughters’ idea. (Seemed happy)

F: What do you think other mothers like Marima can do about Asha’s refusal to get married**?**

P5: They will say, that she is not matured enough to get married, so she shouldn’t get married.

F: Look at this vignette according the tradition of this community, and tell me what women in this community will say to Ms. Marima?

P4: According to this community, if a girl reached the age of 15, she is matured enough to get married. So they will tell her that the girl should get married.

P1: Some people might tell her that the girl has reached 15, so get her married; others might say if a girl got married at the age of 15 she will have trouble while giving birth.

P6: According this community, there is no one who takes the age in to consideration, they will give her for marriage if the boy is capable of feeding the family.

P8: There are some who think that 15 is the right age for a girl to get married, while others think it’s not the right age.

F: Is there any additional comment, or a different one? What do you say?

P7: At this time there is no one who takes the age in to consideration, they will give her for marriage if the boy is capable of feeding the family. So they advise marima to give her for marriage.

F: Two different views are raised here, one says she shouldn’t get married and others say she should. which view is dominant in this area?

P1: The attitude which ‘she should marry’ will take the upper hand.

F: Now we are going to read the vignette from where we stopped, pay attetion. mrs ''Marima'' agreed with 'Asha's '' decission in that Asha should not marry at this age.

F: What would most other mothers those are around her and in community say about Marima with regard to her new idea?

Do you know a girls could not say give me to husband rather the one who wants her to marry ask her.

F: Look! the question says, Mrs. marima took the girl's choice (thought) and decissed this girls should marry. regarding mother's deission what would other mothers say? and how could they see it?

P1: They say ''it is great'' if daughter refuses to marry and her mother confirms that what the daughter asserted and they say it is a splanedid.

F: What additional view could those females [mothers] who have been around could say while they hear what the mother has decided?

P2:Here in our locality when a grl says ''I am not young enough to marry'' other individuals say mariama don't persuade or enforce her.

F: Any additional or new idea, what would other mothers those who have been around or in the community could say while they hear what the mother has decided? just tell what you feel about, please participate. Are you feeling tire?

P4: After marima had said, my doughter is not young enough to marry, they said go out and back later though only 3 years left.

F: hose who take Asha are not around. We still not reached there. What would other mothers say about marima's agreement with her daughter?

P5: They say this is her chance which exactly goes to her and so that they say give her to the one has asked.

P6: Accordng to this locality if some one comes to ask girl for marriage, th entire family to outdoor with the girl which the husband is looking for and make a discussion so that it is not determined by her father only. She is not belongingness goes to neighor's aswell. Today you have no chance to speak, they said a young enough girl for marriage is better if marry. 'Person cant correct himself (Person can't say about himself)'. [Seemed happy]

F: That is good all of you please participate in the form of preverb?

P5There is a differenet between what was happening before and now.

P6: days according to our locality, girls will easly communicate with boys through broker and go together, And they call this democracy. So that these day there is no day one who could talk girls for other boys by being midiator.

P7: In the past if some one says I love your daughter and say i need her for my son and he will that was possibe, And her family or daughter's family would discuss by say can we give them or not?

F: Those who are living in this kebele what would say about marima thought?

P8: Ones your daughter refused marrying at this age no one could have the authority to convince her and they say to marima you have to say she will marry while her age reaches for marriage.

F: It is good here we had two thoughts, the which say she is right, she should not marry and second one i should marry which thought or asumption takes the greater prat?

P2: Your idea is invalid because how dare a mother ask a girl in this way. so that let her marry could take greater number.

P3: Those of you who have additional information what would you say?

P5: The female around here could favor the girls by saying what if you gave birth before marriage.

F: What would the broker say who had been participating on the deal made between Mrs.Marima and Asha?

P2: The negotioter or broker could say the chance which come as a miracle shoud not be let down and so that she should marry.

P5: As the negotioter or broker takes part in the deal he enforce her to marry putting a preasure.

P2: Girls born to be given to strangers she may give birth before marriage at home.

P4: There is no such un mature girl in nature, there is no small girl when we come to marriage.

F: Is there any benefit which negotioter or broker get?

As to me he hasn't, it is favoring whom they know.

F: Would those comments or reactions have an effect on her decission?

P1: Yes, it will let her change her decission.

F: The next question is, is there a situation in which Asha's thought could be acceptable without breaking the norm?

P2: Personally speaking her idea is correct but it is against norm.

F: Is there a condition or circumstance in which Asha's thought could be could be acceptable without breaking the culture?

P4: In the case of this locality a girl whose age is ''15'' is young enough to marry.

P6: No one could see age even if she refused the boy who ame to asked her for marriage, she was not go to school.

P6: Girls couldn't marry whom they don't like.

F: Is the decission Asha became acceptable if she doesn't love the guy?

P7: yes, ineded .

F: Now, if Asha says i don't love the boy (guy) does the family take her assertion forgranted? and do they stop?

P7: They don't stop specially if they know his family indeepth, they wiil't stop.

F1: Is there a sittation in which Ashas decission be acceptable?

P5: Yes, She does need to marry, they can stop anforcing her.
