# Supplementary material for: Adolescent girls trapped in early marriage social norm in rural Ethiopia: A vignette-based qualitative exploration
Source: PLoS One. 2022 Feb 17;17(2):e0263987. doi: 10.1371/journal.pone.0263987 (PMC8853576; doi:10.1371/journal.pone.0263987)
Supplement: S1 File — (ZIP) [file pone.0263987.s002.zip › PDF FGD data/FGD 19_MOAG_D4.pdf]

Mothers of adolescent girl/s_ District 4

Discussants background information		
Name Initial	Education	Age
1) B.A	1	30
2) F.M	Uneducated	30
3) K.L	2	40
4) A.A	1	50
5) W.A	2	50
6) M.A	Uneducated	35
7) M.S	Uneducated	25
8) B.S	2	40

Marriage Vignette

F: What would most other mothers like merima do in this situation?

P2_ Until they make sure the type of person asking they should give a girl of 15 in to youngster. They should refuse until she is of an age.

P4_ Even in the past this was the problem, when the intermediary/delala come they should say my girl is not of age and refuse.

P7_ A child not of age for marriage could face danger during child birth, and the delala may be fooled in to marriage and face problems. Most of the girls between 15 and 18 years of age get married. Even 15, 16, and 17 years of age go after a guy and gets married.

P8_ In our society people are giving girls in to marriage at age of between 18-20. But even at age of 12 they may be given away for marriage, and at the age of 14 and 15 they going in to marriage by themselves.

P5_ In our area there are girls who run after a guy at age of 15 by themselves and returned back by families. But now we have also learned a lesson and the children are getting educated and this trend is stopping. At the age of 17 there are also girls who would run off with a guy. Men get married mostly at age between 20 to 25 years.

P2_ If there is this kind of situation we would let him leave the 15 year girl and let her continue with her studies.

P3_ They will let her get married in this kind of situation.

P1_ If a father lets a girl out of age in to marriage he will be asked by the law.

P1_ If a father forces her to a marriage he will be asked by the law.

F: What would most other mothers advise Merima to do regarding her daughter's refusal to the proposal?

P2_ It will be said to her that the girl is not of age and she should take care.

P7_ Until the desire is hers do not marry her off.

P6_ She will be told not to marry off a refusing girl.

P5_ There are girls that get married leaving behind two older sisters. We will say do not marry off a girl out of age and refusing, she may even bring problems if she get married at this age.

P1_ We will say even the girl is refusing do not force her.

P3_ Even others have created problems when the husband returns her back saying he does not like her anymore, so let your child finish school. Most of the time the mother should not give her permission, nobody is going to say let her get married like her neighbours.

F: Who's going to force Aisha to get married?

P2_ Only the one who's giving her away is the one who can force her.

P1_ The reason could only the one giving her away which means delala and her peers.

P3_ If the mother wants her to get married the neighbor may say girls are born to get married so get married.

F: What would most other mothers say about Merima in this situation?

P3_ We will advise others by saying our girl is not of age. If she was an elder one we would have given her away, but this one is young for marriage and we are going to support the mother on this idea.

P7_ We will support the mother by saying let her be of age.

P6_ We will refuse with the mother.

P5_ Because the mother's idea is right the neighbors will also support her.

P4_ When the girl refuses the neighbours will persuade her by saying girls are born for boys and beg her to get married. If the boy is working and has good income.

F: What would other people such as the delala say about Merima?

P6_ If the mother refuses the marriage broker (delala) will go after the girl and try to persuade her.

P8_ The broker will approach when there is nobody around and tell her that the girl is in love with boy and Mr x has also done the same, you should also should let her get married when the husband is not around.

P1_ The broker may persuade her to run off with boy. He will just send a girl friend and let her run off with the boy.

F: How the adolescent girls in your village get married?

P1_ They will fool her outside; we will only hear that she run off.

F: Would the opinions and reactions of the other mothers make Merima change her mind about her daughter's marriage?

P1_ delala will come back again and again specially to the mother and may persuade her.

P2_ Some people may tell the mother the boy is handsome and try to persuade her.

P3_ The neighbors may put pressure and force her in to marriage.

P4_ People may persuade her, but in the other hand she may not be persuaded because the older one has been returned.

P8_ If they came repeatedly, they told her as the destiny comes once and may be told the boy is smart and wealth, she may change her mind. On the other hand she may the say the older one has been returned so she may refuse.

F: Would their reactions have an effect on here?

P4_ No one is going to pressure her in to giving her to marriage

P8_ There is pressure. Brokers try to persuade her to change her mind, this is also pressure

P2_ No pressure

P1_ If the girl is persuaded it may put pressure on the mother but not effective.

F: if no effect, what makes you think that way?

P6_ Because the daughter's role is important in determining the marriage

P8_ Because it is illegal to force someone into marriage.

F: Are there any circumstances where it would be considered more or less acceptable for Aisha not to get married at her age?

P7_ She can refuse the marriage. She has full right.

P2_ Her refusal has full right, she can refuse. Only her refusal has full right if she doesn’t like the person asked her for marriage

P8_ If she (adolescent girl) wants her refusal to marriage to be accepted, she should firmly say she wants to continue her education. But she cannot refuse by saying she is not of age.

P4_ If the girl refuses no one is going to force her to marry even her father and mother.
