# Supplementary material for: Adolescent girls trapped in early marriage social norm in rural Ethiopia: A vignette-based qualitative exploration
Source: PLoS One. 2022 Feb 17;17(2):e0263987. doi: 10.1371/journal.pone.0263987 (PMC8853576; doi:10.1371/journal.pone.0263987)
Supplement: S1 File — (ZIP) [file pone.0263987.s002.zip › PDF FGD data/FGD 20_UAB_D4.pdf]

Unmarried adolescent Girl_District 4

**Discussants background information**		
Name initial	Education (Grade)	Age
1. AM	4	14
2. CD	8	14
3. AL	8	16
4. CM	8	16
5. KR	8	15
6. AD	5	17
7. LT	5	14
8. AA	8	14

Marriage vignette

I Thank you very much. I will tell you a story of a girl I named Rehima (that is not her actual name) living in this woreda. I would like you to listen to the story carefully and discuss the questions that follow. Rehima is a 16 year old student who lives with her parents. She attends school and helps her mother with household chores. One day Hindiya, Rehima's cousin came over to visit Rehima's family. They are about the same age. Hindiya announces that she is engaged and getting married in a month's time. She also strongly suggests to Rehima that she should also marry soon as she is getting old for marriage. She reveals she also knows that there is someone from their village who is interested in marrying Rehima.

I Is the history clear for you?

All of the discussant as the history is clear and understandable by nodding their head and by saying yes it is clear.

I What would most adolescent girls in Rehima's position do in this situation?

D2 some of the adolescent girls like that of rehima can accept what her friend advice and gets married. Some of them may refuse to accept what hindiya advised and may continue their education.

I Which one is common? The one who continue their education or those who accept and get married?

D3 the one who accepts hindiyas advice is more common even though some of them continue their education.

D4 they tries to convince her saying he had so many things so you better marry him. If you marry him you are the most benefitted one. By doing so her friend plays a great role in diverting her mind to get married.

I Do mostly marry?

D5 mostly accept their advice and get married. They accept hindiyas advice.

All most all of the discussant agreed that they can accept what hindiya advised.

I What would Hindiya and most other girls expect Rehima to do in this situation?

D6 …they expect as she dropout from her education and accept her advice and get married.

D7 she expect as she got married. They feel as she accepts what they advised. Continued saying, some of her friends expect as she continue her education.

I Uhh… other pleases?

D8 most of them expects her to accept and get married.

D1 ….according to this community she tries to convince her to get married.

I But Rehima doesn't want to marry young. She announces that she does not want marry at this age. What would Hindiya and most other girls say about Rehima's decision?

D2 saying, they try to convince her repeatedly.

D3 if she refuses to accept hindiyas advise she can continue her education and may get in good position later.

I Here, she is already decided as she continues her education. She refused their advice. So what did hindiya and her friends react to her?

D3 … leave her. She is mentally ill. She did not accept her friend's advice.

D5 she is foolish. Even she is lazy in her academy. She did not finalize it even she continued now. She returns and get married later.

I Uhh …any other? She said my age is too little.

D6 saying, she is not considered as a human being. Leave her just to continue her education.

D7 occasionally they search a person who convinces her.

I Is there "waltaasiissaa*" means convincer or negotiator*?

D8 *All of the discussant laughed* …uhhhh…and said yes.

D3 he tries to convincing her even by lying her. He told her as if he is in a good state or condition, as if he is wealth enough to lead his life. They also tell her as if it is a good chance for her.

I What if she refuses again?

D4 she is mentally ill. We never talk on her issue other time. Just leave her.

D6 they we will continue our advice.

I Uhh...other please

I What does haaftuu means?

D2 Continues replying as …. *Haftuu* means the one who is not needed in the community.

Her losses the one who marries her even older people. After all she stays at home.

D4 once she refuse our advice, leave her she is *haftuu*. Leave her; she will give birth at home.

D3 she is such kinds of person. Leave her, because she is foolish, never understand what other says. Continue saying she is also considered as the one which is not needed by other as ugly.

D7 a human being can be changed through others idea. After she realizes what they inform, she may say am already remained at home, my friends insult me as such. In this case she may go further and may engage herself even with unknown and older people. Continued saying. She is also decides to hung herself.

I Would the opinions and reactions of her peers make Rehima change her mind about refusing the marriage?

D4 if she was a talented person who does not want to dropout from school, she can manage herself and continue her education. She may not change her decision.

D3 occasionally some person may not change her decision. She can continue her education and may finalize it.

D3 …she changes her decision and engage her to marriage. In our community marriage is highly respected and her (the proposed-to adolescent girl) interest in education would not

matter much. So even if she is not interested to get married, people and peers put pressure on her to get married while somebody shows interest

I What is the decision of most adolescent girls for this situation in this community?

D1 in our community majority of the adolescent girls change their decision and got married.

I Thank you very much. Are there any circumstances where it would be considered more or less acceptable for Rehima not to get married at her age?

D2 if she is a gentle students she can reach where ever she wants.

D3 if she had clear vision she cannot get married.
